# Succeed escape: Flow shear promotes tumbling of *Escherichia coli*near a solid surface

**DOI:** 10.1038/srep35290

**Published:** 2016-10-18

**Authors:** Mehdi Molaei, Jian Sheng

**Affiliations:** 1 Department of Mechanical Engineering, Texas Tech University, Lubbock, Texas, United States of America

## Abstract

Understanding how bacteria move close to a surface under various stimuli is crucial for a broad range of microbial processes including biofilm formation, bacterial transport and migration. While prior studies focus on interactions between single stimulus and bacterial suspension, we emphasize on compounding effects of flow shear and solid surfaces on bacterial motility, especially reorientation and tumble. We have applied microfluidics and digital holographic microscopy to capture a large number (>10^5^) of 3D *Escherichia coli* trajectories near a surface under various flow shear. We find that near-surface flow shear promotes cell reorientation and mitigates the tumble suppression and re-orientation confinement found in a quiescent flow, and consequently enhances surface normal bacterial dispersion. Conditional sampling suggests that two complimentary hydrodynamic mechanisms, Jeffrey Orbit and shear-induced flagella unbundling, are responsible for the enhancement in bacterial tumble motility. These findings imply that flow shear may mitigate cell trapping and prevent biofilm initiation.

The motility of bacteria near surfaces has a broad range of implications, from biofilm formation^1^,^2^,^3^ to biofouling^4^,^5^ and bioremediation of oil spill in environment^6^,^7^,^8^,^9^,^10^,^11^,^12^,^13^. It has been shown that both translational and angular motilities are altered by the presence of a solid surface in a quiescent flow owning to strong hydrodynamic interactions between motile organisms and a solid surface, e.g. reducing surface-normal but increasing surface-parallel swimming speeds^14^,^15^,^16^, reorienting cell body parallel to the surface^17^,^18^,^19^, and swimming in circles^18^, which leads to trapping cells near the surface^17^. Molaei *et al*.^15^ using Digital Holographic Microscope (DHM) and microfluidics have discovered that tumble motility of *E. coli* is strongly suppressed by a solid surface and further shown that hydrodynamic hindrance was the key mechanism in trapping the cell near a surface^15^. The study explained one crucial observation that the formation of biofilm over a solid substrate is promoted regardless cell’s ability to tumble. However, the mechanisms explaining the fact why in nature biofilm is less likely to form over a substrate with flow shear^20^ remains inadequately resolved. This inspired the current investigation.

Flow shear has long been known to modify the motion of bacteria, e.g. Jeffery Orbit (JO) and rheotaxis. Jeffrey orbit^21^ is a periodical motion followed by any aspherical particles embedded in a shear flow, while rheotaxis is considered as motility or behavior responses to flow shear by a motile bacterium^22^ or a swimming micro-organism^23^. In a near surface region, flow shear has profound influence on bacterial motility. Kaya and Koser^24^ have shown that non-motile bacteria in a shear flow simply follow Jeffery Orbits near a surface but at slower angular velocity in comparison to that in bulk. Additional upstream migration due to near surface shear is also reported^25^. In a free shear flow, Marcos *et al*.^22^,^26^ have shown that due to the chirality of flagella, the bacteria migrate in the direction normal to the plane of shear, namely rheotaxis. Locsei and Pedley^27^ using theoretical analysis has demonstrated that correlation of the swimming direction before and after a tumble is disrupted by intrinsic Jeffrey Orbit (JO) motions. When cells are subjected to a linearly varying shear, Zottl and Stark^28^,^29^ using simulations and Rusconi *et al*.^30^ accompanied with experiments have shown that motile micro swimmers like bacteria perform periodic swinging, and result in helical-like trajectories. This helical like trajectory can be disrupted due to the flexibility of bacterial flagella and a small amount of a random angular reorientation^31^. These studies have shown that the complex interplays between intrinsic JOs and shear gradients provide hydrodynamic mechanisms to suppress the bacterial transport in the direction of shear gradient and to trap cells in high shear region. Rusconi *et al*.^30^ further develop this concept that this shear-induced trapping mechanism would enhance cell-surface collision and lead to the higher rate of surface attachment. Conversely, Chilukuri *et al*.^32^ have articulated that since the near surface flow shear preferably aligns the cell in the flow direction, i.e. particles in shear flow spend large fraction of time in the streamwise direction, it reduces the probability of bacterium-surface collisions. The supporting observations are reported by^20^. However, this inconsistency highlights the current lack of understanding of the effects of flow shear on the near-surface bacterial motility, especially the motility relevant to cell orientation.

Here, we applied DHM and microfluidics^15^,^33^ to study the effects of flow shear on the motility of wild-type *E. coli* (AW405) when it swims near a solid surface. We have succeeded in using DHM to simultaneously image up to ~8000 wild-type *E. coli* bacteria over the entire 200 μm depth of a microfluidic device, with a spatial resolution of 0.2 μm (lateral) and 0.5 μm (axial). By enabling simultaneous tracking of a large number of cells without any moving parts in the setup, this approach establishes DHM as a powerful technique for studying the motility change in the presence of environmental stimuli.

## Results and Discussion

**Figure 1:**
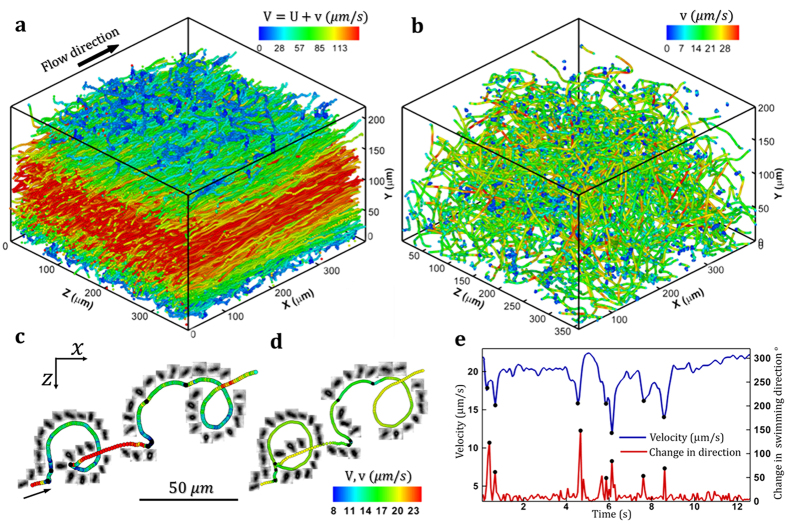
Sample swimming trajectories of wild-type *E. coli* (AW405) in a moderate shear flow (Surface shear, S = 3.0 s^−1^). (**a**) Sample 3D trajectories (2000 out of 8345) presented in a Eulerian laboratory frame of reference. Color-code: the magnitude of the absolute velocity (*V*) composed of the flow advection (*U*) and bacterial swimming velocity (*v*), (**b**) Bacterial swimming trajectories (same in (**a**)) in a Lagrangian frame of reference. Color-code: bacterial swimming velocity, *v*. A sample 3D trajectory superimposed by in-focus reconstructed bacterial images (only every seven images are shown) in (**c**) Eulerian, and (**d**) Lagrangian coordinates. (**e**) Sample bacterial swimming speed (Blue) and change in swimming direction vs. time. Black dots shown in (**c–e**) mark the detected tumble events.

A sample of DHM trajectories of intermediate shear (S = 3 s^−1^) in a fixed frame of reference is shown in Fig 1a (only 2000 of 8345 trajectories are displayed and color-coded with the swimming speeds). In bulk, since flow advects faster than bacterial swimming, trajectories are shown as straight lines with the perceivable fluctuations. While these straight lines represent mean advection by the flow, the fluctuations are the relative motions generated by bacterial swimming. Note that the swimming speed is orders of magnitude larger than that of Brownian dispersion. To investigate bacterial swimming motility independent from the flow advection, the relative motion (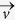) is extracted by subtracting the local flow motion, 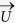, from the Eulerian *E. coli* velocity, 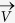 (details in SI §S1.5). The corresponding 3D Lagrangian trajectories (viewed from a fluid particle initiated at the same starting position as each bacterium) are reconstructed and shown in Fig. 1b. A sample trajectory superimposed with in-focus reconstructed bacterial images (only shown every seven frames) is shown in Fig. 1c and its corresponding Lagrangian trajectory in Fig. 1d. Both 3D position and angular orientation of individual bacterium are accurately obtained. For brevity unless specified directly, bacterial motilities (swimming and tumbling) refer to those obtained from Lagrangian trajectories only. To separate effects of flow shear from those of a solid surface^15^, swimming statistics, especially tumble motility, will be studied in the same near surface region but at four different flow conditions characterized as surface flow shear (*S* = 0,0.06,3, and 30 s^−1^). To be consistent with prior study on surface suppression on tumble motility^15^, we define near surface region as a volume within 20 *μm* (or 2 cell body length, *L*_*c*_, including flagella) to the nearest surface (0 ≤ *y* ≤ 20 *μm* and 180 ≤ *y* ≤ 200 *μm*).

**Figure 2:**
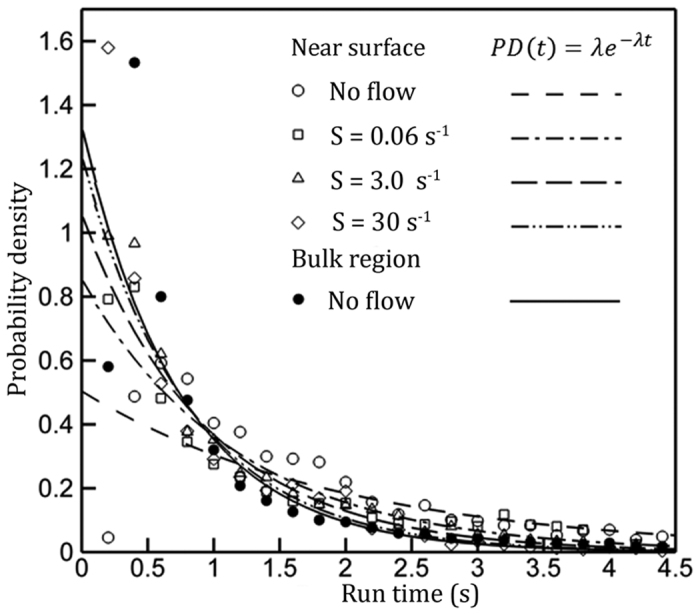
Probability density (PD) distributions of the mean runtime (symbols) and least square fits of the exponential model (lines), *PD*(*t*) = *λe*^−*λt*^, where *λ* is the characteristic tumble frequency 15,34. Solid symbols: bulk measurement; hollow symbols: mean runtime measurements in the near surface region (0 ≤ *y*/*L*_*c*_ ≤ 2 or 0 < *y* < 20 *μm*) under flow shear.

Anecdotally shown in Fig. 1c,d, in spite of flow shear *E. coli* demonstrates general swimming behavior as it would near a solid surface in quiescent condition, e.g. swimming in circles interrupted by tumbling events (black dots on the trajectory), and run-tumble. However, additional swimming pattern specifically attributable to shear flows such as rheotaxis, i.e. cell migrates in the direction normal (*z*) to the shear (*x-y*) plane is also observed (Fig. 1c,d). This rheotaxis behavior has previously been reported for bacteria^22^ and for bi-flagellated green algae^23^. In this paper, we focus on tumble motility and characterize a tumble as a temporal event with concurrent reduction in swimming speed and sudden large angle change in the swimming direction (Fig. 1e, Refs 15 and 34), hence decompose each trajectory into a series of sequential run and tumble events. Table 1 summarizes near surface motility change of *E. coli* (AW405) in the presence of flow shear. Flow shear near a surface has no effect on swimming speed which remains 6~9% higher than that in the bulk^15^. However, the near surface mean run time (inverse of tumbling frequency) decreases as flow shear increases, from*T*_*s*_ = 1.94 ± 1.96 s at no flow shear to *T*_*s*_ = 0.91 ± 0.71 s at 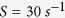. Recall that in a quiescent flow, Molaei *et al*.^15^ have shown that a solid surface prolongs *T*_*s*_ and reduces the tumble frequency. Statistics in Table 1 reveals that the flow shear assists bacteria to overcome hydrodynamic hindrance imposed by a solid surface and promotes tumbling of *E. coli*. Note further that the trends of tumbling angle and linear dispersion (*D*) with respect to flow shear corroborate very well with the abovementioned assertion (Table 1). This trend can be further shown with probability density (PDs) of run time at different flow shears in the near surface region (symbols in Fig. 2). In the absence of shear, PD has a pronounced tail with a peak at *T*_*s*_ = 0.6*s*, while as the flow shear increases, the tail of PD reduces and peak approaches to 0.2 *s*. The mean tumbling frequencies are estimated by finding the e-folding timescales, *λ*, through the best fit of exponential distribution of *λe*^−*λt*^ (Ref. 34, lines in Fig. 2), i.e. tumble frequencies were λ = 0.5, 0.85, 1.05 and 1.23 tumbles/s in correspondence to the near surface shear rates, S = 0, 0.06, 3 and 30 *s*^−1^ respectively. Clearly, one can conclude that contrary to the surface’s suppression effect on tumbles, flow shear near it promotes the tumble motility of *E. coli.,* by reducing mean run time with the increase of shear.

**Figure 3:**
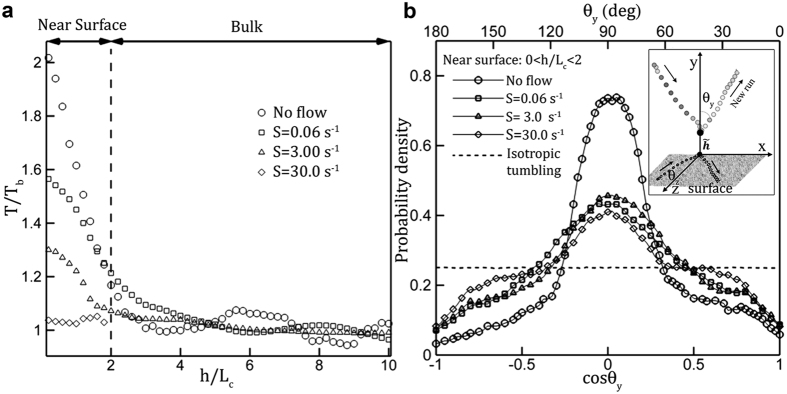
Near surface flow shear mitigates surface induced quenching of tumbling and surface-normal motility. (**a**) Mean runtime, *T*, at different distances from the surface, *h*, normalized with the total cell length, *L*_*c*_, at different flow shears in comparison to that in bulk region in a quiescent flow, *T*_*b*_. Near-surface mean runtime, *T*/*T*_*b*_, is reduced as near surface flow shear, *S*, increases. Symbols: DHM measurements averaged over a 5 *μm* thick, surface-parallel layers. (**b**) PDs of the cosine of the exit angle, *θ*_*y*_, i.e. the orientation of a new run immediately after a tumble to the surface normal direction (illustrated in Inset). Symbols: Statistics of surface normal motility collected over a 20-μm thick surface parallel layer. Dashed line: isotropic distribution of *θ*_*y*_.

Flow shear’s promoting and surface’s suppressing effects on tumbles extended 20 μm into the fluid, equivalent to 2*L*_*c*_. This was determined by computing the mean run time, *T*, at different distances from the surface, *h*, over 5 μm thick layers parallel to the surface (Fig. 3). In the absence of flow shear, *T* peaked at the surface and regressed hyperbolically to the value in the bulk, *T*_*b*_, (Ref. 15, circles in Fig. 3a). The similar regressive relation of *T*/*T*_*b*_ with respect to *h*/*L*_*c*_ were also observed at shear less than 30 *s*^−1^, while the distribution almost reduced to a flat line at high shear (*S* ≥ 30 *s*^−1^). As flow shear increases, the rate of regression decreases (Fig. 3a), i.e. the effect of shear on promoting tumble increases. This is consistent with other statistics, e.g. distributions of *T*_*s*_ and *λ* with respect to flow shear.

Molaei *et al*.^15^ has further shown that in a quiescent flow, a solid surface preferentially suppressed tumbles in the direction normal to the surface by hydrodynamic hindrance. In current study, we have inspected the effect of flow shear on tumbling angle (the change in direction between two consecutive runs). The PD measured in cosine of tumbling angle with respect to y-axis (*θ*_*y*_, defined in inset of Fig. 3b) shows that in the near surface region, the tumble angle deviates substantially from the uniform distribution and forms a narrow peak at cos *θ*_*y*_ = 0^15^, as the flow shear increases the peaks widen and peak values reduce. To accentuate effects, we have composed our statistics and PDs over a layer of 20 μm distance from the surface (0 < *h*/*L*_*c*_ < 2, Fig. 3b). Briefly, a solid surface restricts the tumble within the surface parallel layer, while the near surface shear allows cell to tumble easily in the surface normal direction that improves with flow shear.

The results (Figs 2 and 3, Table 1) clearly show that contrary to surface induced suppression^15^, near a sheared solid surface tumble becomes an effective mechanism for cells to escape from the solid surface and reduces the cell concentration near a surface (Fig. S3). These results also provide a plausible complimentary mechanism that may prevent biofilm from initially forming over a surface in the presence of flow shear.

**Figure 4:**
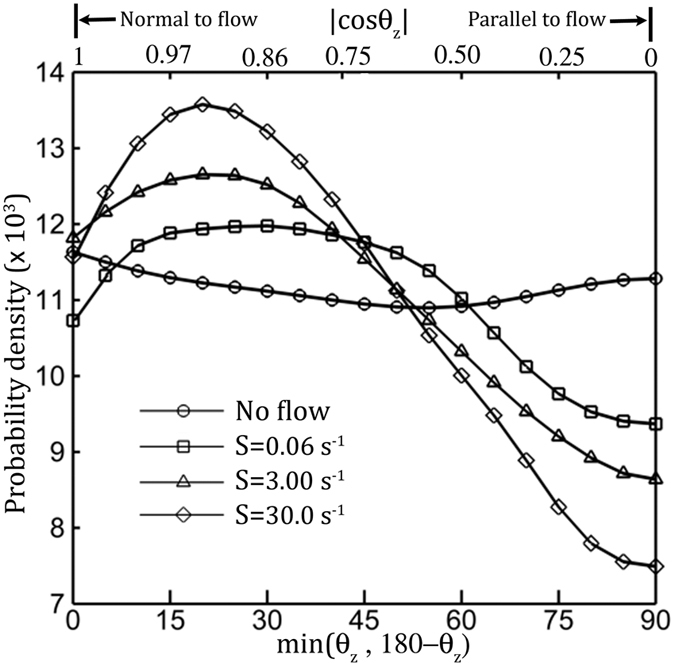
Flow shear causes motile *E. coli* to rheotaxis, i.e. swim in the direction normal to the flow. PDs of mean swimming direction during a run, *θ*_*z*_, i.e. the orientation of an run immediately before a tumble, to cross-flow direction (z axis), are collected over a 20-μm thick surface parallel layer. To include rheotaxis in both positive and negative z axis, we used the acute angle, min(*θ*_*z*_,180° − *θ*_*z*_) to compose PDs with the bin size of Δ*θ*_*z*_ = 10^*o*^ and at an interval of 5°.

What are the mechanisms by which flow shear promotes bacterial tumble near a surface? Two key mechanisms that cause cells to reorient in a shear flow over a surface, i.e. Jeffrey Orbit motion by aspherical particles and tumble by active swimmers, are considered. The former (JOs) describes periodic reorientation of the fore-aft axis of an aspherical particle in a shear flow with the period of 
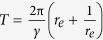
. where *r*_*e*_ is the aspect ratio of the particle, and 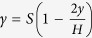 is the local flow shear. Ruscini *et al*.^30^ show that smooth swimming bacterial strain (*B. subtilis*) undergos JOs and *r*_*e*_ is determined as the length of the entire cell including flagella (~10 *μm*) versus the width of cell body (~1 *μm*). Kaya *et al*.^24^ further demonstrate, using a non-motile strain of *E. coli*, that bacteria in a shear flow near a solid surface also undergo JOs at a period calculated by a modified *r*_*e*_ that took into account of hydrodynamics of cell, shear, and surface. It is conceivable that such a reorientation could be interpreted as a tumble if this reorientation satisfies the tumble criteria, i.e. the change in cell orientation exceeds 55° in less than 1/15 s and low swimming speed^15^,^34^. We have performed numerical simulations to examine this mechanism and showed that with adequate flow shear, JOs can generate large enough change in the swimming direction within the time normally allotted to execute a tumble event (details in SI §S2). For brevity, we refer to this mechanism as pseudo-tumble. Further analysis shows that the cut-off shear needed for current strain (AW405, *r*_*e*_ = 10) to perform pseudo-tumble must exceed 18 *s*^−1^ (Figs S8 and S9). Although pseudo-tumble contributes to enhancement of tumble motility at high shear regimes (*S* > 18 *s*^−1^), this mechanism utterly fails to explain enhancement in tumble motility (Figs 2 and 3) at low shear regimes (*S* < 18 *s*^−1^) due to its exclusion of other important mechanisms, e.g. tumble and wobble as well as rheotaxis by swimming bacteria (Refs 26,35 and 36, Fig. 4).

**Figure 5:**
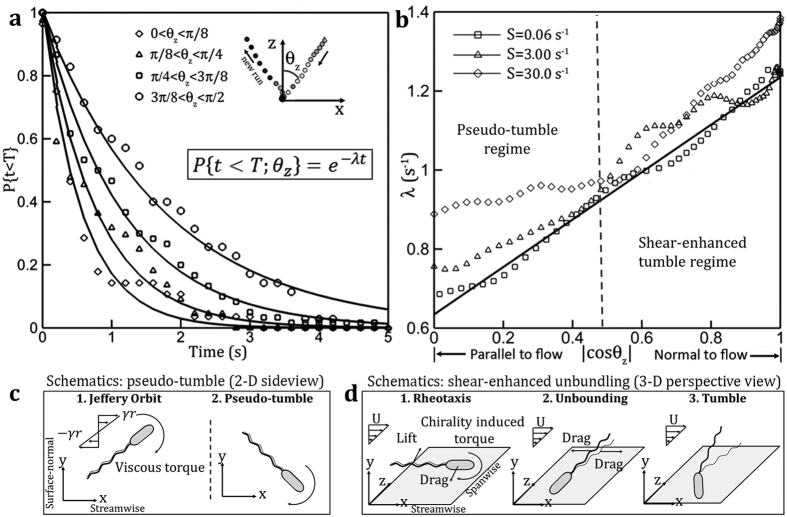
Flow shear promotes flagella unbundling, resulting in enhanced near surface tumble frequency. (**a**) Sample survival probability distributions (symbols) of near surface run time at flow shear (S = 0.06 s^−1^) conditionally sampled based on the mean run orientation immediately before a tumble to the cross-flow, *θ*_*z*_ and least square fit (solid lines) of exponential model, *e*^−*λt*^, where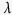 is the characteristic tumble frequency. Each distribution was conditionally sampled from runs over evenly distributed bins with a size of 22.5° (or *π*/8). (**b**) Profiles of characteristic tumble frequency, λ, vs. the cosine of *θ*_*z*_ at different flow shear of S = 0.06 s^−1^ (squares), 3.0 s^−1^ (triangles), and 30 s^−1^ (diamonds) respectively. Each data point was calculated by estimating *λ* from the ensemble survival probability distributions conditionally sampled over uniform bin size of 22.5° and centered at an interval of 2° Schematics of two dominant cell reorientation mechanisms near a solid surface with flow shear: (**c**) Kinematic sequences elucidating the passive mechanism of “pseudo” tumble when *E. coli* swims in the direction of flow: Step 1 – An spheroid experiences Jeffery Orbit (JO) motion in a shear flow; Step 2 – when flow is sufficiently large, the reorientation by JO motion is rapid enough to be considered as a tumble. (**d**) Sequences showing shear enhanced flagella unbundling mechanism that results in enhanced tumbling frequency in the direction normal to the flow: Step 1 – Chirality induced torque orients the cell in the direction normal to the shear plane, a.k.a. rheotaxis; Step 2 – local flow shear promotes flagella unbundling; Step 3 – cell executes a tumble.

In the following, we propose a complimentary mechanism based on the effect of flow shear on the unbundling process during a tumble to the cell re-orientation mechanism in shear flow by Jeffery Orbit (Fig. 5c). Elucidated graphically in Fig. 5d, the mechanism can be described: As a bacterium swims in a shear flow, a moment due to cell’s chirality causes it to reorient and to swim preferentially in the direction normal to the shear (*x-y*) plane, a process also known as rheotaxis^26^,^35^,^36^ (left panel in Fig. 5d). When a bacterium swims normal to the shear plane, shear flow causes additional drag forces on the unbundling flagellum (thin filament) and flagella bundle (thick filament in Fig. 5d). They act normal to filament axes but in opposite directions, and differ substantially in magnitudes (middle panel in Fig. 5d). Compounded with the existing viscous forces during unbundling in the quiescent condition, these additional drag forces will accelerate the unbundling process and promote a tumble (right panel in Fig. 5d). Note that the proposed mechanism can only reduce the time scale needed to unbundle but is less likely to affect the time scale of other tumbling processes by Denton *et al*.^37^. Hence, it is not expected to alter the statistics of tumble motility in bulk, i.e. tumbling frequency and mean run time (Table 1). While in the near surface region the tumble motility without flow shear is shown to be strongly suppressed due to the hydrodynamic hindrance of flagella unbundling^15^, flow shear near a surface enhances the flagella unbundling and counteracts surface originated hydrodynamic hindrance as long as the flagella bundle is aligned normal to the shear plane. Hence, the proposed mechanism improves tumble frequency and reduces the mean run time, which provides us with a plausible explanation of tumble enhancement in low shear flow regimes (Figs 2 and 3). The additional analysis to compare the effects of these two hydrodynamic mechanisms: shear accelerated unbundling and surface hydrodynamic hindrance, on the unbundling process is further provided in SI S2.4.

Note that flow shear cannot affect the unbundling if the flagella bundle is aligned in the shear plane. One can argue that to have the abovementioned mechanisms to generate pronounced effects on tumble motility (Figs 2 and 3), two necessary premises must occur and can be verified: (i) At near surface region with flow shear, a large number of cells must swim in the z direction (at least at a shallow angle). This will allow the deviates from uniform distribution of cell population in cell orientation necessary to bias the tumble statistics; (ii) Runs in the cross-flow (*z*) direction must contain shorter runs than those aligned in the flow (*x*) direction. The mean statistics of runs must demonstrate linear dependency on a cosine of the direction of each run to z axis, *θ*_*z*_. In the following, we employ conditional sampling over near surface measurements to validate that these two necessary conditions are indeed satisfied, and consequently support the proposed mechanism.

The PDs of mean direction of each run at different flow shear rate are computed over the acute angle between the mean run direction to the *z* direction, min(*θ*_*z*_, 180° − *θ*_*z*_), for simplicity, *θ*_*z*_, hereinafter (Inset in Fig. 4). At no flow shear (circle in Fig. 4), the PD is uniform and suggests an isotropic cell alignment and random swimming direction. As the shear increases, bacteria are increasingly aligned with *z* axis, shown as a peak at 20° with a decreasing width, and the number of cell aligned in the *x-y* plane (*θ*_*z*_ = 90°) is substantially reduced. This phenomenon of preferential swimming by microbes in shear flows, known as rheotaxis, has been widely reported for bacteria^26^,^35^,^36^ and for biflagellate green algae^23^, but first for peritrichous bacteria, Our measurement of rheotaxis velocity, *v*_*z*_, on the tumble capable strain of *E. coli* (AW405), agrees well with results of smooth swimming bacteria species (Ref. 22, Fig. S3). Clearly, hydrodynamic interaction of flow shear on cell body reorients the cell in cross-flow direction, setting the stage for the shear assisted unbundling mechanism to take effect.

To verify the second hypothesis, i.e. runs in the cross flow direction are shorter than those aligned in flow direction, we perform ensemble sampling of runs over near surface trajectories conditioned on mean *θ*_*z*_ of each run. The PDs are obtained within a sampling bin of Δ*θ*_*z*_ centered at *θ*_*z*_, and characteristic tumbling frequency, *λ*, can be estimated with best fit (Fig. 2). To avoid fluctuations in conditionally sampled data and improve accuracy, we estimate *λ* using the survival probability, 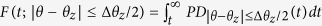, where 
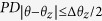
 is PD of runs within the sampling bin centered at *θ*_*z*_. Since PD assumes the exponential distribution, *F*(*t*) assumes the form of *F*(*t*) = *e*^−*λt*^. The analysis is applied to cases of all flow shears and sample result (S = 0.06 ^−1^) is presented in Fig. 5a. The *λ*s are approximated by best fit of *F*(*t*) collected within four sampling bins with a bin size of 22.5° centered evenly over the first quadrant. The results confirm our assertion that at *S* = 0.06 s^−1^, bacterial swimming in z direction (diamond in Fig. 5a) tumbles more frequently than those aligned in the *x* direction. This analysis is applied to estimate *λ*s with respect to *θ*_*z*_ at all shear cases, and the results are plotted with respect to cos (*θ*_*z*_) as Fig. 5b. In low flow shear regimes (*S* < 18 *s*^−1^), the *λ* shows an expected linear relation of cos (*θ*_*z*_), since only the projection of flow shear normal to the flagella can affect the unbundling time scale and consequently tumble frequency. As *S* increases beyond a critical shear, the profile of *λ* shows linear relationship for cos (*θ*_*z*_) > 0.5 (or *θ*_*z*_ < 60°), but a substantially elevated deviation for those cell aligns primarily in the direction of flow (*θ*_*z*_ 60°). This increase in *λ*s suggests that this enhancement is generated by pseudo-tumbles. The profile (diamond in Fig. 5b) clearly demonstrates that two primary mechanisms causing cells to reorient in a near-surface shear flow are taking effects at the same time.

## Conclusions

The run and tumble motility of bacteria plays crucial roles in many microbial processes. Focusing on tumbling motility, i.e. a key mechanism for angular dispersion of bacteria, the paper aims to elucidate complex hydrodynamic interactions between bacteria and its environmental stimuli, e.g. flow shear and surface. Using the system composed of *E. coli* and a solid surface under shear flows, we have demonstrated that subtle interplay of hydrodynamic forces on flagella in the presence of a solid surface and flow shear could significantly alter motility and subsequently change the dispersion characteristics of bacteria suspension near a surface. Using DHM and microfluidics platform, Molaei *et al*.^15^ has clearly shown that near a solid surface in a quiescent flow, reduction in hydrodynamic forces among flagella during a tumble event disrupts the flagella unbundling process, and reduces the tumbling frequency as well as limits the angular dispersion. Consequently, this tumble suppression mechanism by hydrodynamic surface interaction causes cells to be trapped near a surface, and increases the probability of cell attachment.

In this paper, we demonstrated extensively that contrary to the case in Ref. 15, the flow shear can alleviates the suppression of tumbles and promotes angular dispersion near a solid surface, and subsequently reduces cell concentration as well as lowers the probability of cell attachment. This effect on “tumble” motility is shown to be effective throughout all shear regimes, via two diametric but complimenting mechanisms. In the high shear regime (*S* > 18 *s*^−1^), the flow shear will reorient bacteria due to their being aspherical, by the mechanism known as Jeffrey Orbits. As the shear is sufficiently large, the reorientation is rapid and large enough to be considered as a tumble. This is however a passive mechanism native to any elongated cells regardless of their motility, but is only effective at high shear rate for those cells preferably aligned in the flow direction or in the shear plane. Contrary to this well accepted mechanism, we demonstrate in this paper that the additional mechanism does exist that is effective for motile *E. coli* at all shear rates. This mechanism is based on the conjecture that the flow shear normal to the flagella bundle during a tumble accelerates the unbundling process and hence alleviates the unbundling suppression via the same hydrodynamic interaction brought-on by a solid surface^15^. This is a mechanism effective only for motile *E. coli* swimming out of the plane of shear and having a clear cosine dependency of swimming direction to shear normal direction. The statistical significance of this mechanism is greatly magnified by bacterial rheotaxis. These two mechanisms provide a plausible explanation on why a surface with running water is less likely to initiate a biofilm in contrast to the mechanism of shear erosion on a fully developed biofilm, and further emphasize the importance of hydrodynamics in microbial motility, especially tumble motility near a surface.

## Methods

### Experimental setup

A straight polydimethylsiloxane (PDMS) channel was used (45 mm length, 200 μm depth (H), and 5 mm width. See details in Ref. 15). The sample area was sufficiently far from the entrance of the channel to ensure the fully developed flow in the *x* direction, and the large width-to-height ratio ensured the dominate flow shear located in the *x-y* plane. The bacteria suspensions were driven by a syringe pump (NE-1000, New Era pump System Inc.). The studies were conducted under three centerline velocity, *U*_max_, of 3, 150 and 1500 *μm*/*s* (or maximum shear rate located at both surfaces of 0.06, 3, and 30 *s*^−1^, respectively). As shown in Fig. S2, the flow velocity profile, *U*_*x*_(*y*), is parabolic and agrees well with the solution of a 2D Poiseuille flow, as
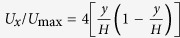
. The advection velocity reached its maximum at the center, *y* = 100 *μm* and the local flow shear, *γ*, varys linearly with y as 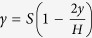, where the maximum shear are located at both surfaces, 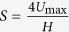


### Digital Holographic Microscopy

We applied DHM^38^ with correlation based de-noising technique^33^ to image and track bacteria in three dimensions. The detail of the technique and DHM is fully documented in Ref. 33. The digital holograms were recorded at 15 fps by a 2048 × 2048 CCD camera (Imprex-4ML) and a transmission microscope (TS-100) at the magnification of 40X (Plan Fluor 40X Objective, NA = 0.65). The measurement volume is 400 × 200 × 400 μm^3^. We succeeded in applying DHM to simultaneously image up to ~8000 of *E. coli* in a shear flow over the 200 *μm* depth, with spatial resolutions of 0.2 μm (lateral) and 0.5 μm (axial) in a span of one minute.

### Data analysis

To accurately characterize the bacterial motility (best described in Lagrangian frame of reference) from trajectories (experimentally measured in the laboratory frame of reference), the advection flow is measured directly by applying μPIV analysis to reconstructed holographic bacterial images at a given depth, and later applied frame-to-frame Particle Tracking Velocimetry (PTV) analysis to improve the accuracy of near surface measurement (SI S1.4). Although motile cells are often ineffective flow tracers due to their swimming motion, the coherent motion among those cells at same depth in a microfluidics must be the advection generated by the flow (streamwise velocity profiles, *U*(*y*), along y axis are shown in Fig. S2). Once the flow field, *U*(*y*), is approximated, the swimming speed, 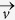, of a cell in Lagrangian frame of reference is calculated by subtracting the local flow velocity, 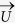
(*y*), from the measured cell velocity in Eulerian coordinate, 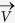, i.e. 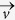
(*t*) = 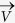
(*x*, *y*, *z*; *t*) − 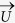
(*y*). The Lagrangian swimming trajectory and cell positions are determined by 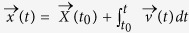, where 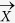
(*t*_0_) is the initial position of the trajectory at the starting time, *t*_0_. Briefly, the physical interpretation of 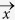
(*t*), is a trajectory measured from a frame reference fixed on a fluid particle initiated at same starting position as the bacterium, 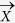
(*t*_0_). The motility analysis (detailed in Ref. 15) is then applied to Lagrangian trajectories from thereon.

### Culture

*E. coli* AW405 were saturate grown in LB medium over time, re-cultured in tryptone medium and then prepared by washing the cell in motility buffer. Details on culture and sample preparation procedures are provided in SI S1.1.

## Additional Information

**How to cite this article**: Molaei, M. and Sheng, J. Succeed escape: Flow shear promotes tumbling of *Escherichia coli* near a solid surface. *Sci. Rep.* **6**, 35290; doi: 10.1038/srep35290 (2016).

## Supplementary Material

Supplementary Information

## Figures and Tables

**Table 1:** Motility of wild-type *E. coli* in near-surface region at different flow shears, compared to results in the bulk. The tumbling angle is the angle between two consecutive runs.

		Flow shear (s^−1^)	No. of bacteria	Mean speed (*μms*^−1^)	Run time (s)	Tumbling angle (deg)	Tumble freq. *λ* (s^−1^)	Dispersion, *D* (10^−9^ m^2^ s^−1^)	Dispersion anisotropy		
									*A*_*xx*_	*A*_*yy*_	*A*_*zz*_
Molaei, *et al*. (2014)	in bulk	0	2194	14.1 ± 8.0	0.93 ± 1.32	71.3 ± 44.0	1.3	0.2	−0.03	−0.14	0.17
	near surface	0	556	15.3 ± 6.8	1.94 ± 1.96	46.7 ± 39.1	0.5	0.14	0.57	−0.93	0.36
This study		0.06	2115	15.9 ± 6.4	1.28 ± 1.38	72.6 ± 41.0	0.85	0.11	0.33	−0.72	0.39
		3.00	2775	15.6 ± 8.2	1.07 ± 1.03	71.5 ± 37.2	1.05	0.09	0.51	−0.73	0.22
		30.0	1035	14.9 ± 8.3	0.91 ± 0.71	94.7 ± 33.3	1.23	0.05	−0.17	−0.90	1.07

*D*_*ii*_(*i* = *x*, *y*, *z*) are the dispersion coefficients along the three Cartesian directions (*y*: surface-normal direction), computed from the autocorrelation of the Lagrangian swimming velocity, *v*, *D* = (*D*_*xx*_ + *D*_*yy*_ + *D*_*zz*_)/3 is the mean dispersion coefficient, and 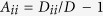 measures dispersion anisotropy. The number of trajectories used to compile statistics are listed in the second column, and represent mean ± standard deviation.

## References

[b1] Costerton J. W., Stewart P. S. & Greenberg E. P. Bacterial biofilms: A common cause of persistent infections. Science 284, 1318–1322 (1999). 10334980 10.1126/science.284.5418.1318

[b2] Dalton T. . An *in vivo* polymicrobial biofilm wound infection model to study interspecies interactions. PLoS One 6 (2011). 10.1371/journal.pone.0027317 PMC3208625 22076151

[b3] Vaccari L. . Films of bacteria at interfaces: three stages of behaviour. Soft Matter 11, 6062–6074 (2015). 26135879 10.1039/c5sm00696a

[b4] Holmstrom C., Egan S., Franks A., McCloy S. & Kjelleberg S. Antifouling activities expressed by marine surface associated Pseudoalteromonas species. FEMS Microbiol. Ecol. 41, 47–58 (2002). 19709238 10.1111/j.1574-6941.2002.tb00965.x

[b5] Yebra D. M., Kiil S. & Dam-Johansen K. Antifouling technology - past, present and future steps towards efficient and environmentally friendly antifouling coatings. Prog. Org. Coat . 50, 75–104 (2004).

[b6] Acosta-Gonzalez A., Rossello-Mora R. & Marques S. Characterization of the anaerobic microbial community in oil-polluted subtidal sediments: aromatic biodegradation potential after the Prestige oil spill. Environ. Microbiol. 15, 77–92 (2013). 22626032 10.1111/j.1462-2920.2012.02782.x

[b7] Beazley M. J. . Microbial community analysis of a coastal salt marsh affected by the deepwater horizon oil spill. PLoS One 7 (2012). 10.1371/journal.pone.0041305 PMC3399869 22815990

[b8] Diaz E., Jimenez J. I. & Nogales J. Aerobic degradation of aromatic compounds. Curr. Opin. Biotechnol. 24, 431–442 (2013). 23122741 10.1016/j.copbio.2012.10.010

[b9] Fuchs G., Boll M. & Heider J. Microbial degradation of aromatic compounds - from one strategy to four. Nat. Rev. Microbiol. 9, 803–816 (2011). 21963803 10.1038/nrmicro2652

[b10] Kleinsteuber S., Schleinitz K. M. & Vogt C. Key players and team play: anaerobic microbial communities in hydrocarbon-contaminated aquifers. Appl. Microbiol. Biotechnol. 94, 851–873 (2012). 22476263 10.1007/s00253-012-4025-0

[b11] Kostka J. E. . Hydrocarbon-degrading bacteria and the bacterial community response in Gulf of Mexico beach sands impacted by the deepwater horizon oil spill. Appl. Environ. Microbiol. 77, 7962–7974 (2011). 21948834 10.1128/AEM.05402-11 PMC3208977

[b12] McGenity T. J. Hydrocarbon biodegradation in intertidal wetland sediments. Curr. Opin. Biotechnol. 27, 46–54 (2014). 24863896 10.1016/j.copbio.2013.10.010

[b13] McGenity T. J., Folwell B. D., McKew B. A. & Sanni G. O. Marine crude-oil biodegradation: a central role for interspecies interactions. Aqua. Biosys . 8, 10 (2012). 10.1186/2046-9063-8-10 PMC3465203 22591596

[b14] Frymier P. D., Ford R. M., Berg H. C. & Cummings P. T. 3-Dimensional tracking of motile bacteria near a solid planar surface. Proc. Natl. Acad. Sci . 92, 6195–6199 (1995). 7597100 10.1073/pnas.92.13.6195 PMC41669

[b15] Molaei M., Barry M., Stocker R. & Sheng J. Failed escape: Solid surfaces prevent tumbling of Escherichia coli. Phys. Rev. Lett. 113 (2014). 10.1103/PhysRevLett.113.068103 25148353

[b16] Ramia M., Tullock D. L. & Phanthien N. The Role of Hydrodynamic interaction in the locomotion of microorganisms. Biophys. J. 65, 755–778 (1993). 8218901 10.1016/S0006-3495(93)81129-9 PMC1225777

[b17] Berke A. P., Turner L., Berg H. C. & Lauga E. Hydrodynamic attraction of swimming microorganisms by surfaces. Phys. Rev. Lett. 101 (2008). 10.1103/PhysRevLett.101.038102 18764299

[b18] Lauga E., DiLuzio W. R., Whitesides G. M. & Stone H. A. Swimming in circles: Motion of bacteria near solid boundaries. Biophys. J. 90, 400–412 (2006). 16239332 10.1529/biophysj.105.069401 PMC1367047

[b19] Li G. . Accumulation of swimming bacteria near a solid surface. Phys. Rev. E 84 (2011). 10.1103/PhysRevE.84.041932 22181200

[b20] McClaine J. W. & Ford R. M. Reversal of flagellar rotation is important in initial attachment of Escherichia coli to glass in a dynamic system with high- and low-ionic-strength buffers. Appl. Env. Microb . 68, 1280–1289 (2002). 10.1128/AEM.68.3.1280-1289.2002 PMC123756 11872478

[b21] Jeffery G. B. The motion of ellipsoidal particles immeresed in a viscous fluid. Proc. R. Soc. Lond. A 102, 161–179 (1922).

[b22] Marcos Fu H. C., Powers T. R. & Stocker R. Bacterial rheotaxis. Proc. Natl. Acad. Sci. 109, 4780–4785 (2012). 22411815 10.1073/pnas.1120955109 PMC3324032

[b23] Chengala A., Hondzo M. & Sheng J. Microalga propels along vorticity direction in a shear flow. Phys. Rev. E 87 (2013). 10.1103/PhysRevE.87.052704 23767563

[b24] Kaya T. & Koser H. Characterization of hydrodynamic surface interactions of Escherichia coli cell bodies in shear flow. Phys. Rev. Lett. 103 (2009). 10.1103/PhysRevLett.103.138103 19905544

[b25] Kaya T. & Koser H. Direct upstream motility in Escherichia coli. Biophys. J. 102, 1514–1523 (2012). 22500751 10.1016/j.bpj.2012.03.001 PMC3318139

[b26] Marcos Fu, H. C., Powers T. R. & Stocker R. Separation of Microscale Chiral Objects by Shear Flow. Phys. Rev. Lett. 102 (2009). 10.1103/PhysRevLett.102.158103 19522552

[b27] Locsei J. T. & Pedley T. J. Run and Tumble Chemotaxis in a Shear Flow: The Effect of Temporal Comparisons, Persistence, Rotational Diffusion, and Cell Shape. Bull. Math. Biol. 71, 1089–1116 (2009). 19198954 10.1007/s11538-009-9395-9

[b28] Zottl A. & Stark H. Nonlinear dynamics of a microswimmer in Poiseuille flow. Phys. Rev. Lett. 108 (2012). 10.1103/PhysRevLett.108.218104 23003306

[b29] Zottl A. & Stark H. Periodic and quasiperiodic motion of an elongated microswimmer in Poiseuille flow. Eur. Phys. J. E 36 (2013). 10.1140/epje/i2013-13004-5 23321716

[b30] Rusconi R., Guasto J. S. & Stocker R. Bacterial transport suppressed by fluid shear. Nat. Phys . 10, 212–217 (2014).

[b31] Tournus M., Kirshtein A., Berlyand L. V. & Aranson I. S. Flexibility of bacterial flagella in external shear results in complex swimming trajectories. J. R. Soc. Interface 12 (2015). 10.1098/rsif.2014.0904 PMC4277081 25376876

[b32] Chilukuri S., Collins C. H. & Underhill P. T. Impact of external flow on the dynamics of swimming microorganisms near surfaces. J. Phys.-Condes. Matter 26 (2014). 10.1088/0953-8984/26/11/115101 24590066

[b33] Molaei M. & Sheng J. Imaging bacterial 3D motion using digital in-line holographic microscopy and correlation-based de-noising algorithm. Opt. Express 22, 32119–32137 (2014). 25607177 10.1364/OE.22.032119 PMC4317141

[b34] Berg H. C. & Brown D. A. Chemotaxis in Escherichia coli analysed by three-dimensional tracking. Nature 239, 500–504 (1972). 4563019 10.1038/239500a0

[b35] Marcos & Stocker R. Microorganisms in vortices: a microfluidic setup. Limnol. Oceanogr. Methods 4, 392–398 (2006).

[b36] Rusconi R. & Stocker R. Microbes in flow. Curr. Opinion Microb . 25, 1–8 (2015). 10.1016/j.mib.2015.03.003 25812434

[b37] Darnton N. C., Turner L., Rojevsky S. & Berg H. C. On torque and tumbling in swimming *Escherichia coli*. J. Bacteriol. 189, 1756–1764 (2007). 17189361 10.1128/JB.01501-06 PMC1855780

[b38] Sheng J., Malkiel E. & Katz J. Digital holographic microscope for measuring three-dimensional particle distributions and motions. Appl. Optics 45, 3893–3901 (2006). 10.1364/ao.45.003893 16724155

