# Corrigendum: Succeed escape: Flow shear promotes tumbling of *Escherichia coli* near a solid surface

**DOI:** 10.1038/srep42567

**Published:** 2017-02-22

**Authors:** Mehdi Molaei, Jian Sheng

Scientific Reports 6: Article number: 3529010.1038/srep35290; published online: 10 18 2016; updated on: 02 22 2017

The Acknowledgements section in this Article is incomplete.

“Research was supported in part by NSF under the grant no. CBET-1341901, NIH under the grant no. 1-R21-EB008844-01, and GoMRI (Gulf of Mexico Research Initiatives) under the grant no. SA12-03/GoMRI-003”.

should read:

“This research was made possible in part by a grant from The Gulf of Mexico Research Initiative (Grant No. SA12-03/GoMRI-003), and in part by grants from NSF (Grant No. CBET-1341901) and NIH (Grant No. 1-R21-EB008844-01). Data are publicly available through the Gulf of Mexico Research Initiative Information & Data Cooperative (GRIIDC) at https://data.gulfresearchinitiative.org (doi:10.7266/N7445JJQ)”.

