# Supplementary material for: Succeed escape: Flow shear promotes tumbling of Escherichia colinear a solid surface
Source: Sci Rep. 2016 Oct 18;6:35290. doi: 10.1038/srep35290 (PMC5082759; doi:10.1038/srep35290)
Supplement: Supplementary Information [file srep35290-s1.pdf]

Supplemental Information: Succeed escape: Flow shear promotes tumbling of *Escherichia coli* near a solid surface

Mehdi Molaei[*ǂ], Jian Sheng[*ǂǂ]

[*]Department of Mechanical Engineering, Texas Tech University, Lubbock, Texas

[ǂ]Current: Department of Chemical and Biomolecular Engineering, University of Pennsylvania, Philadelphia, Pennsylvania

[ǂǂ]Current: Department of Mechanical Engineering, Texas A&M University, email: jian.sheng@tamucc.edu

S1 Experimental Method

S1.1 Bacterial culture

E.coli AW405were prepared by obtaining cells from a single colony on an agar plate which was used to inoculate 10ml of LB medium. The culture was incubated by an orbital shaker (Model 980184, Talboys) over night at 30 °C while shaking at 120 rpm. When the culture reached to the saturation growth (OD_{600}=1), 100 μl of the solution is inoculated with10 ml of tryptone medium following with incubating at 35 °C and shaking at 120 rpm for 4 hours, until the optical density reached to OD_{600}=0.45. The cultured bacterial suspension was then transferred into a 10ml vial and was placed in a centrifuge at 2000 rpm to be washed. After carefully removing supernatant from the vial, the cells were suspended in a motility buffer (10^{-2} M potassium phosphate buffer; pH = 7.5) containing 10^{-4} M EDTA. This process was repeated twice to gain the best motility behavior of the cells. The prepared bacterial suspension was then transferred to a syringe to be injected in a microchannel. To create the shear flow inside the microchannel, bacterial solution was driven by a syringe pump (NE-1000, New Era pump System Inc.) to create constant flow rate inside the microchannel. The flow rates tested in the experiment were in the range of $0.2 - 100\ \mu l/min$.

S1.2 Microfluidics

The microchannel (45 mm long, 200 μm deep, and 5 mm wide) used in the experiment was fabricated following soft lithography techniques [2]. The photoresist used for microchannel molds, SU-8 2150 (MicroChem Corp) were spin coated on a silicon wafer and baked on a hot plate to deposit a layer with 200 μm thickness. The channel feature was transferred to photoresist by soft-

lithography [2]. Polydimethylsiloxane (PDMS; Sylgard 184 Silicone Elastomer Kit, Dow Corning) was cast on the master and cured in the oven at 70°C for 6 hours. After curing, microchannel was bonded with bonded with a clean microscopic glass slide with anti-reflection (AR) MgF_2 coating (<1% at 500-670nm) using ozone plasma activation. The bonding however temporary lasted for 10 hours during the entire experiments. We used the AR coated glass slide to reduce the coherent noise in holograms caused by the internal reflection among optics.

S1.3 Cell imaging and tracking

Imaging is performed using inline digital holography (DHM, [3-10]) on an inverted microscope (TS-100, Nikon) with 40X magnification (Plan Fluor 40X Objective, NA=0.65, Nikon). Briefly, the optical setup for DHM includes a laser beam (7 mW He-Ne, 25LHR171-249, Mellosgriot-CVI), Neutral Density filter (ND = 2), a spatial filter, and a collimating lens. Digital holograms are recorded by a CCD camera (Imprex-4ML) at 15fps. The raw holograms are de-noised and enhanced using the correlation based de-noising technique described in details [11]. The field of view of the imaging is 400 × 400 μm^2 with reconstruction depth of 200 μm, and with a spatial resolution of 0.2 μm (lateral) and 0.5 μm (axial). After recording and reconstructing of the holograms we track the bacteria for 1 minute using in-house software to obtain bacterial position, velocity, and trajectories. To determine the angular motion of the cell during tumbles, we need to identify tumble events within trajectories. Two criteria are considered to determine a tumble event. The first criterion is a fast change in the swimming direction and it is satisfied when the angle between two consecutive swimming directions is more than 50 degree. The second criterion is an abrupt change in the swimming speed and it is satisfied when the swimming speed dropped more than one standard deviation below the mean swimming speed of each individual cell. The time between two consecutive tumbles was considered as a run time. The swimming direction before a tumble were computed over four consecutive positions of the cell prior to the tumble and the direction after the tumble were computed over four positions after the tumble. The tumble angle was the angle between these two direction considered as a tumbling angle. Note that the abovementioned motility analysis was only applied to bacterial trajectory with the flow advection velocity removed.

S1.4 Shear flow measurement by micro-Particle Image Velocimetry (μPIV) and Particle Tracking Velocimetry (PTV)

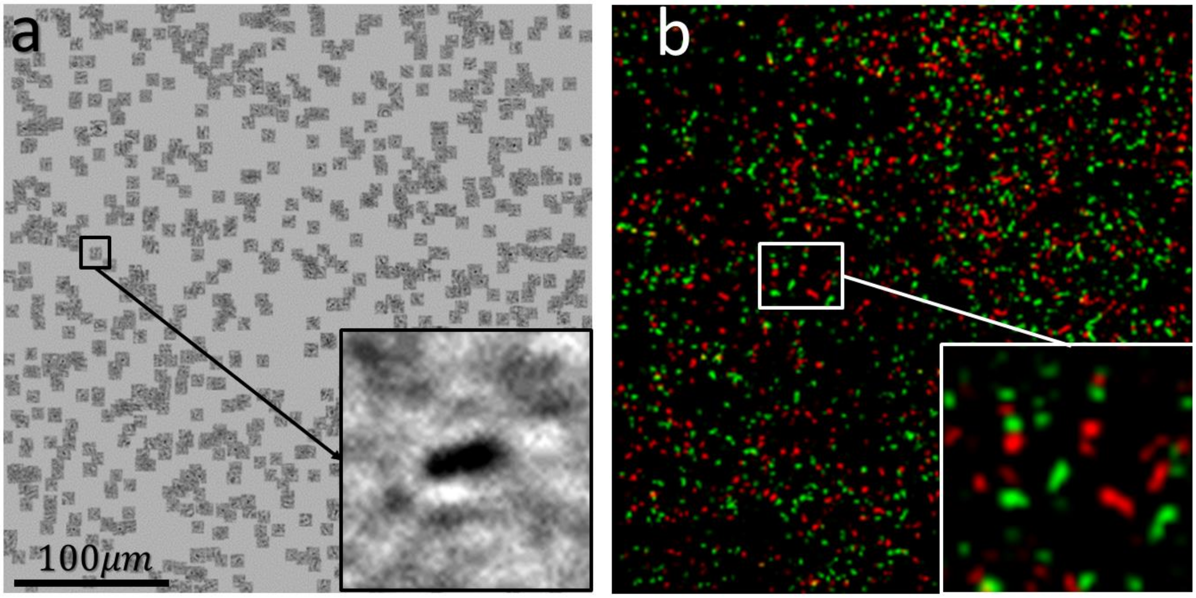

Fig. S1| (a) In-focus images of *E.coli* recorded at 50 randomly selected time steps. The cells in the images are located in the mid-plane of the channel with thickness of 2.5μm *(98.75μm<y_c<101.25 μm)*. (b) Green spots: first PIV image obtained by applying intensity inverting and a proper threshold on the image shown in (a).The original PIV image used for analysis is in gray scale; however, white pixels are replaced by green to clarify the image. Red spots: second PIV image obtained from images recorded with times lapse of $dt = 2/15\ s$ with respect to first PIV image.

To estimate flow velocity within the channel, we performed μPIV analysis to the reconstructed holographic bacterial images at different depths of the channel to obtain mean velocity distribution. Concerns may arise whether bacteria cells would be an appropriate flow tracer since they are motile. In the absence of mechanical stimuli, the bacteria move randomly hence their spatially averaged motion must vanish. The coherent motion among those cells at the same depth in the microchannel yields the advection generated by the flow. Additional particle tracking procedure (PTV) are applied to near surface cell motion to further improve the near surface flow estimation. In the following paragraph, we present the PIV analysis including constructing PIV images from a stack of reconstructed holographic images. We then show PTV analysis for near surface flow measurement.

μPIV analysis: Cross-correlation based PIV analysis is applied to a sequence of reconstructed 3D particle fields to obtain mean advection flow profile along the depth direction (y axis). To determine the flow velocity at a given y position, PIV image pair, $I(x, z; y, t_1)$ and $I(x, z; y, t_2)$

where t_1 and t_2 are separated by a small time delay, is constructed using in-focus images of bacteria located at that depth using the following ensemble averaging procedure:

$$I(x,z;y,t) = \min_{y}\{C(x,z;y_c,t) | |y_c - y| \leq b_y/2\} \quad \text{(S1)}$$

where $C_i(x,z;y_{c_i},t)$ is the *xz*-plane light scattering of the *i-th* bacterium at its in-focus depth, y_{c_i}, and reconstructed at time frame, t; and b_y is the thickness of the layer within which all in-focus cell images will be integrated. The "ensemble averaging" procedure employed here is a simple minimization operation along the y axis (denoted as the subscript, y, under the operator, min, in Eqn. S1), since the in-focus bacterial image are darker than their surroundings. Note that to allow high the measurement accuracy in a wall bounded shear flow, the integration thickness, b_y, must be kept as small as possible, e.g. $b_y = 2.5\mu m$ in current experiments. This constraint prevents forming the PIV image with high tracer density for any single time step. We circumvent the problem by superimposing particle images, $I(x,z;y,t)$, of a series of time steps, randomly selected over the entire sequence for cross-correlation analysis:

$$I_1(x,z;y) = \min_{t}\{I(x,z;y,t) | t \in (t_1 \dots t_N)\},$$

$$I_2(x,z;y) = \min_{t}\{I(x,z;y,t) | t + dt \in (t_1 \dots t_N)\}, \quad \text{(S2)}$$

where t is the subset of the entire recordings and $t + dt$ is the same randomly selected subset but with a specific time delay, dt. Note that the integration is still performed using minimization along the time axis. Fig. S1a represents an example of I_1 located in the middle of the channel, *y=100μm*, with the bin size of $b_y = 2.5\mu m$. The image is integrated over 50 randomly selected 3D DHM particle fields out of 20,000 recordings. To perform cross-correlation PIV analysis, PIV images obtained in Eqn. (S2) must be inverted (as illustrated in Green or Red color fields in Fig. S2). Fig. S1b shows the superimposed image pair of I_1 and I_2 at a time lapse of two time frames. The same in-focus image of cells, I_1, (shown in Fig. S1a) are inverted, thresheld, and represented as green dots in Fig. S1b. The second PIV image with a small time lapse of $dt = \frac{2}{15}s$ is shown as red dots in Fig. S1b. Once an image pair is obtained, the 2D mean velocity distribution (U_x and U_z in the *x* and *z* directions respectively) at the given depth, y, is determined using FFT-based cross-correlation PIV analysis. Since the flow in microfluidics is often assumed homogeneous along the streamwise (x) direction and the dimension of the sample area in the spanwise (z) direction is much smaller that the channel width, the spatial variations in the obtained velocity field is expected to

be small. For simplicity, only averaged streamwise velocity component, $U_x(y)$, will be retained. The abovementioned technique will be applied to the image pairs at various depth locations, to obtain the velocity profile, $U_x(y)$, along the y direction.

Estimating flow velocity accurately using motile bacteria as tracer particles requires advection flow to be coherent and bacterial motility to be random in directions. However, due to the potential bias error introduced by averaging over insufficient data points (e.g. angular distribution of bacteria is not uniformly distributed over the range of $[0, 2\pi]$), the relative error can be reduced where the local advection velocity, U_x, is much larger than the swimming speed, v. In bulk, the flow advection is larger than the swimming speed of the cells, spatial averaging yields accurate results. In near-surface region, the swimming speed of the cell is larger than the flow velocity; the bias error is often overwhelming. To resolve the problem, we further perform Particle Tracking Velocimetry (PTV) after conventional PIV analysis to improve the measurement accuracy in near-surface flow velocity. We performed PTV near the surface where flow velocity was smaller than swimming velocity i.e. $y \leq \frac{H}{2}\left(1 - \sqrt{1 - \frac{v}{U_{max}}}\right)$. Briefly describe the PIV assisted PTV algorithm below:

(i) Extract the position of a bacterium, (x_p, y_p, z_p), at time frame, *t*;

(ii) Compute streamwise advection velocity from velocity profile, $(U_x(y_p), 0, 0)$, obtained from PIV analysis above and subsequently the displacement due to flow advection;

(iii) Obtain the position of the same bacterium at time frame, $t + dt$, by searching the list of 3D bacterial positions at $t + dt$, around the position estimated by flow, $\left(x_p + U_x(y_p) \cdot dt, y_p, z_p\right)$ and subsequently particle velocity, $V_p(t)$.

(iv) Repeat Step i-iii until all bacteria in the near surface regions are processed.

Once particle velocity is obtained for a time step, the near surface velocity profile, $\vec{U}(y)$, is recomputed by binning V_ps with a size of 1μm at the interval of 1μm and averaging over each bin and over time, $\vec{U}(y) = < \vec{V} >$, where <> denotes average operator over time and each bin. The measured velocity profile, $U_x(y)$, is fitted with a 2D Poisseulle flow profile. Fig. S2 shows the velocity profiles for three different flow shears normalized by their corresponding centerline velocities with the measurement accuracy as error bars.

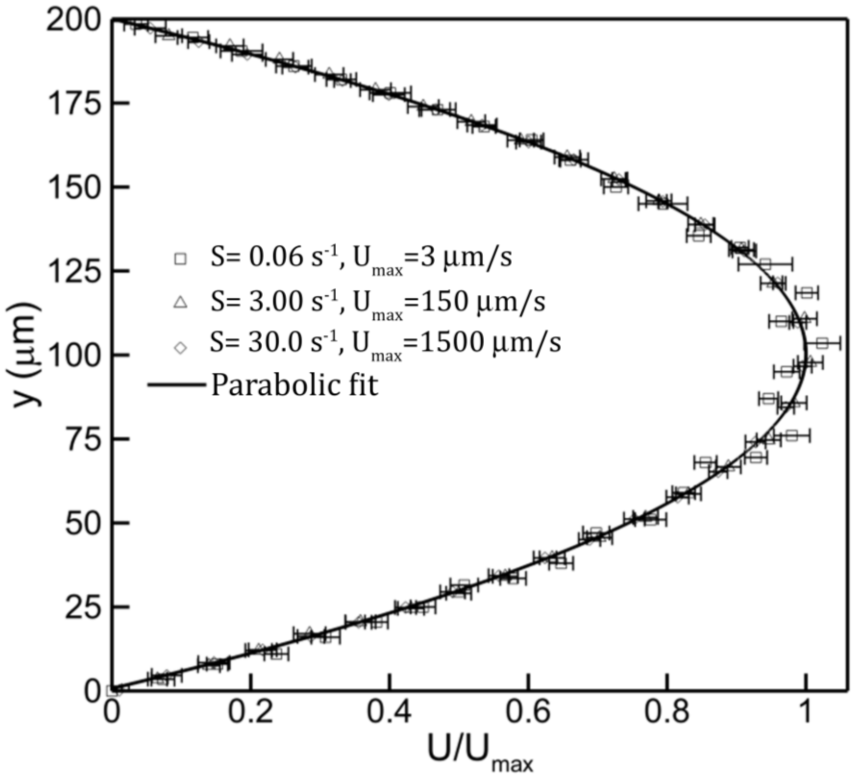

Fig. S2| Normalized flow velocity profile, $U_x(y)$, along the depth (y) direction with the velocity at the centerline velocity, U_{max}. Symbols: measurements averaged over 1 μm thick surface parallel layers. Error bars: one standard deviations. The solid line is the least square fit of a 2D Poiseuille flow over measurements.

S1.5 Determination of Lagrangian bacterial swimming motility

Once the flow advection, $U_x(y)$, and bacterial trajectories are obtained using procedures in (S.1.4 and S.1.5), the Lagrangian swimming motility, i.e. the swimming motion viewed from the moving frame of reference fixed on a fluid particle released at the same location as the bacterium cell (or in short the motility with the flow advection removed) need to be obtained. All analyses are applied to the Lagrangian motilities.

To transform trajectories obtained a Eulerian coordinate, $\vec{X}_p(t)$, to a Lagrangian coordinate, $\vec{x}_p(t)$, we will first measure the swimming speed of a cell, $\vec{v}_p(t)$, by subtracting the local flow advection, $\vec{U}(\vec{X}_p(t), t)$, where y_p is the y position of the cell, p, at time, t, from the measured swimming velocity, $\vec{V}_p(t)$:

$$\vec{v}_p(t) = \vec{V}_p(t) - \vec{U}(\vec{X}_p(t), t). \qquad \text{(S3)}$$

Note that the advection velocity in current experiment contains only one non-zero component in the streamwise (x) direction, $U_x(y)$. Subsequently, the Lagrangian trajectory, $\vec{x}_p(t)$, is determined as the following,

$$\vec{x}_p(t) = \vec{X}_p(t_0) + \int_{t_0}^{t} \vec{v}_p(t)dt. \qquad \text{(S4)}$$

Fig. 2b shows the sample Lagrangian bacterial trajectories and the same trajectories in Eulerian coordinates (Fig. 2a). A sample trajectory in Eulerian coordinates (Fig. 2c) and its Lagrangian trajectory (Fig. 2d) are superimposed with the cell's in-focus images.

Rheotaxis velocity:

When a bacterium with helical flagella swim in a shear flow chiral force acting on the flagella coupled with drag force on the cell body leads to reorienting the cell normal to the shear plan. The lateral drift velocity depends on shear rate and swimming speed [12]. Our experimental data for rheotaxis velocity of wild type *E.coli* agrees with results of smooth swimming bacteria species. Fig. S3 shows the rheotaxis velocity normalized with swimming speed of *E.coli* AW405 and mathematical model provided by Marcos *et al.* [12] for wild type and smooth swimming bacteria.

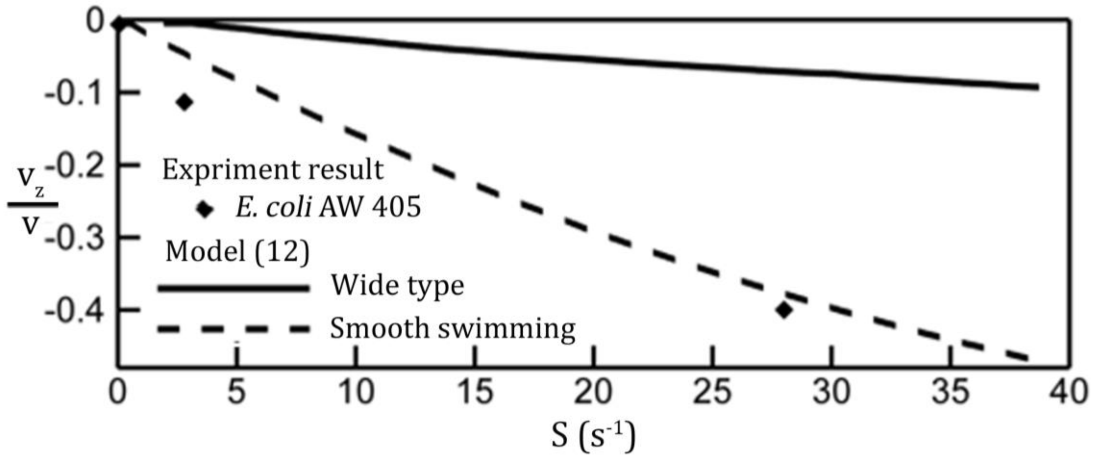

Fig. S3|. The rheotaxis velocity, v_z, plotted relative to the mean swimming speed, v, as a function of the shear rate. Diamonds: DHM measurements for wild-type strain AW405. Curves: mathematical model from Marcos *et al.* for a wild type bacterium (solid line) and a smooth swimming bacterium (dashed line).

S1.6 Profiles of cell concentration We bin the cell positions obtained from each hologram into 5μm thick layers parallel to the surface of the channel at an interval of 2μm. We compute the Instantaneous cell concentration profile, $n(y,t)$ at time step, t. The time mean concentration profile along the depth (y) direction is calculated by averaging the instantaneous concentration profiles over 1 minute of recordings (900 realizations) for each flow condition. The concentration profiles are normalized by the average concentration within a 20um thick surface parallel layer in the middle of channel, $n_0 = < n(y,t) | 90 \le y \le 110\mu m >$, where $< >$ is the average over time and depth.

The normalized concentration profiles at flow surface shear rates, S, are shown in Fig. S4. The solid lines are fitted curves based on the model, $\frac{n(y)}{n_0} = exp\left[L\left(\frac{1}{y} + \frac{1}{H-y}\right)\right]$, by Berek *et al.* [1], where H = 200 μm is the channel depth, n_0 is the cell concentration in the bulk, and L is the characteristic accumulation length scale. Inset figure shows the values of L decreasing with increasing of flow

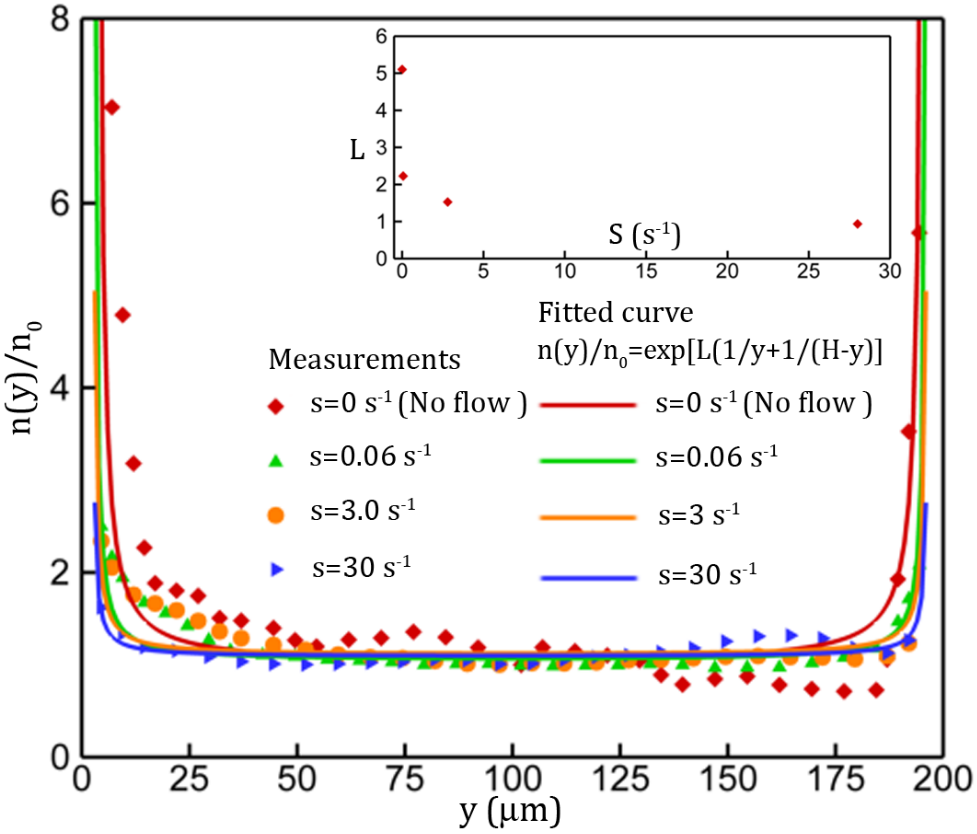

Fig. S4| Concentration of *E.coli* (AW405) as a function of distance, y, from the bottom surface. Symbols: Measurements, Solid lines: Best fit based on the model $\frac{n(y)}{n_0} = exp\left[L\left(\frac{1}{y} + \frac{1}{H-y}\right)\right]$ 1. Color: Flow shear at 0, 0.06, 3.0 and 30.0 s^{-1}, respectively. Inset shows the characteristic accumulation length scale

shear, S. The results reveal that high surface shear leads to less cell accumulation near a solid surface and promotes surface normal dispersions in comparison to those near a surface without flow shear.

S1.7 Characteristic tumbling frequency

Method: The characteristic tumbling frequency is estimated directly from the run time statistics. Two methods are used: (i) estimation of e-folding time using the probability density (PD) distribution of the run time distribution; (ii) estimation of e-folding time using the survival probability of the run time distribution. Both methods are based on the same statistics and should yield the same results, but with the different sensitivities to fluctuations in probability density distributions.

Direct estimation using PDs of the run time distribution: When the PDs are smooth, e.g. in the case of distributions of mean run time within the near surface region like Fig. 2, the PDs are assumed to be exponential, $PD(t) = \lambda e^{-\lambda t}$ [13]. The best fits of $PD(t)$ measurements yield the characteristic tumble frequencies at different shear rate (Fig. 2).

Direct estimation using the survival distribution: When the PDs are fluctuating due to lack of sufficiently large data point when constructing the statistics, such as conditional sampled mean run time based on the swimming orientation to the spanwise (z) direction (Fig. 5a and Fig. S5). In this case, direct estimation using the prior method will yield large errors. Instead, we estimate the e-folding time scales using the survival probability derived directly from the PD,

$$F(t) = 1 - \int_0^\infty PD(t)dt \qquad \text{(S5)}$$

The e-folding time scales can be obtained by fitting Eqn. S5 with the exponential function, $e^{-\lambda t}$. The accumulative probability in Eqn. S5 provide a smooth distribution for accurate estimation.

As mentioned in the main text, we bin the run time of each run in a near surface region based on its mean run angle with respect to the spanwise (z) direction, θ_z. Each bin has a size of $\pi/8$ and two adjacent bins are separated at $\pi/8$. The sample survival functions were plotted in Fig 5a ($S = 0.06\ s^{-1}$), and Fig. S5 ($S = 3$ and $30\ s^{-1}$, respectively) and their best fits of exponential distribution.

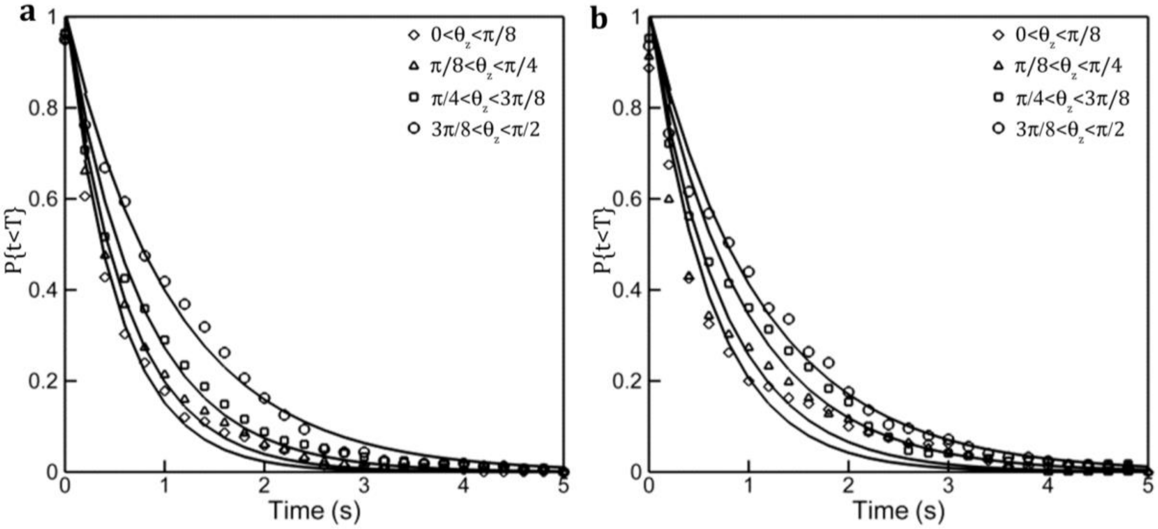

Fig. S5| Survival probability based on the run time data collected within four sampling bins with a bin size of *π/8*. (a) $S = 3.0\ s^{-1}$, and (b) $S = 30.9\ s^{-1}$. Symbols: Measurements of survival probability by cumulative integration of probability density. Lines: best fits of the exponential function, $e^{-\lambda t}$.

Brief recap: Noted that both methods yield similar distributions, i.e. exponentially decrease as the run time increases (Fig. 5a and Fig. S4). It is also noted that a larger characteristic tumble frequency, λ, has a faster decay rate. Elucidated clearly in Fig. 5a and Fig. S5, at any flow shear, when *E. coli* swims in the cross-flow (z) direction, the motile cell is more likely than those swim in the streamwise (x) direction. The tumbling motility decreases as the swimming direction with respect to z axis increases.

S2 Bacterial reorientation in a shear flow: *tumble* and *pseudo-tumble*

S2.1 Hypothesis on cell re-orientation

Our experimental results clearly show that when wild type *E. coli* (tumble capable strain, AW405) swims over a solid surface in the presence of flow shear, the suppressed tumble motility [11] is strongly mitigated and the characteristic tumble frequency, λ, is increased with the near surface flow shear, *S* (Fig. 2). It is found that this effect depends highly on cell orientation, i.e. when *E. coli* swims normal to the flow (i.e. at acute angle to the z axis), the characteristics tumble frequency, λ, is larger than that when it swims in the direction of flow (Fig. 5a, Figs S5a and S5b). It is also shown clearly in Fig. 5b that at low flow shear cases ($S = 0.06, 3\ s^{-1}$), λs increase linearly with respect to the magnitude of $|\cos(\theta_z)|$, where θ_z is the swimming angle of each run

to z axis; whereas at high flow shear case ($S = 30.9\ s^{-1}$), we observe a noticeable deviation from linear distribution at the alignment towards the flow direction, i.e. $|cos\theta_z| = 0$.

As discussed in main text, two mechanisms are considered capable of explaining this shear-induced re-orientation enhancement: In the swimming direction normal to the flow, the near surface flow shear ($S > 0$) helps the flagellar unbundling and improve tumble motility; while in the swimming direction in the flow direction, the sufficiently large near surface flow shear (above a critical shear) will cause non-spherical particle to re-orient passively via Jeffrey Orbit and rapidly enough to cause a large sudden change in the swimming direction, of which it can be mistakenly considered as a "pseudo" tumble. We have demonstrated adequately in the main text that the shear-induce tumble mechanism is indeed a key alternative to the well accepted passive mechanism (e.g. Jeffrey Orbits). In the section, we will explore that at sufficiently high flow shear, the passive mechanism (Jeffrey Orbit) will play increasingly significant role in cell re-orientation.

S2.2 Simulation of reorientation based on Jeffrey Orbit of passive particle

We use a mathematical model for passive prolonged particle in a free shear flow with the characteristic shear, S[14,15] to represent the swimming bacteria. The characteristic length scales used in model are estimated based on the entire cell including cell body and flagella. The objective of this simulation is to first verify whether the angular motion of the cell due to flow shear (Jeffery Orbit), can lead to a large enough reorientation angle during a characteristic time scale of a tumble event, i.e. 1/15s in current case.

The linear and angular kinematics of a smooth swimming *E. coli* can be modeled as the following set of linear dynamic equations:

$$\dot{x} = v sin\theta_z sin\phi \quad \text{(S6)}$$

$$\dot{y} = v sin\theta_z cos\phi \quad \text{(S7)}$$

$$\dot{z} = v cos\theta_z \quad \text{(S8)}$$

$$\dot{\phi} = S\left(1 - \frac{2y}{H}\right)(1 + G cos2\phi) \quad \text{(S9)}$$

$$\dot{\theta}_z = S\left(1 - \frac{2y}{H}\right)\frac{G}{2} sin2\theta_z sin2\phi \quad \text{(S10)}$$

$$G = \frac{r_e^2 - 1}{r_e^2 + 1} \quad \text{(S11)}$$

where v is the swimming speed, and S is surface shear on the bottom surface of the microfluidics with the channel height of H; the parameter, G, is the geometry factor (Eq. S11) based on the aspect ratio of the cell, r_e [16] including flagella. We define the location of the cell as the position vector, $\vec{r} = x\vec{e}_x + y\vec{e}_y + z\vec{e}_z$, where $\vec{e}_x$, $\vec{e}_y$ and $\vec{e}_z$ are unit vectors in the x, y and z directions, respectively; and the orientation of the cell as the unit vector of the cell's fore-aft axis, $\vec{e}_b = sin\theta_z sin\phi\ \vec{e}_x + sin\theta_z cos\phi\ \vec{e}_y + cos\theta_z \vec{e}_z$, where θ_z is the angle between $\vec{e}_b$ and $\vec{e}_z$, and ϕ is the angle between the projection of $\vec{e}_b$ in the *x*-*y* plane, $\vec{e}_b - (\vec{e}_b \cdot \vec{e}_z)\vec{e}_z$ and $\vec{e}_y$. For instance, when $\theta_z = 0$, the cell is oriented normal to the shear (*x*-*y*) plane; and where $\theta_z = 90$, the cell lies in the shear (*x*-*y*) plane. The evolution of linear and angular vector, $\vec{r}(t)$ and $\vec{e}_b(t)$, are obtained by numerical integration of the coupled dynamic equations (S6-10) with the 4th order Runge-Kutta scheme with initial conditions, $\vec{r}(0)$ and $\vec{e}_b(0)$. The change in cell orientation over the characteristic elapse time of a tumble event, $\Delta t_{tumble} = 1/15s^{-1}$, is then measured as the angle between the orientation, $\vec{e}_b(t + \Delta t_{tumble})$ and $\vec{e}_b(t)$. If this change is larger than 50° (mean angle change during a tumble event provided by Berg & Brown [13],, we consider the occurrence of a "pseudo" tumble event.

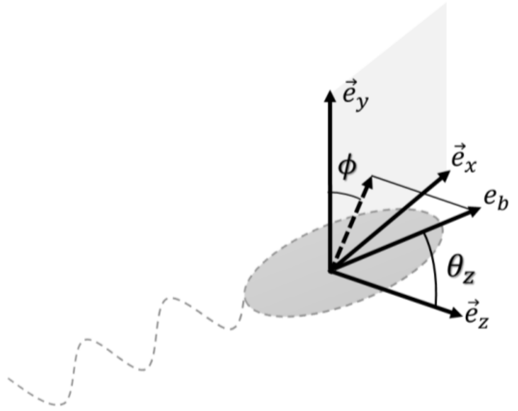

Fig. S6|: definition of $\vec{e}_b$ unit vector of the cell's fore-aft axis, θ_z the angle between $\vec{e}_b$ and $\vec{e}_z$, and ϕ is the angle between the projection of $\vec{e}_b$ in the *x*-*y* plane, $\vec{e}_b - (\vec{e}_b \cdot \vec{e}_z)\vec{e}_z$ and $\vec{e}_y$.

Cell statistics					Flow		Initial conditions		
Width (μm)	Length (μm)	r_e	G	v ($\mu m/s$)	S (s^{-1})	H (μm)	y (μm)	ϕ	ϕ
1	10	10	0.98	15	30	15	5	$\pi/4$	$\pi/4$

Table S1: Conditions used in the simulation shown in Fig. S5.

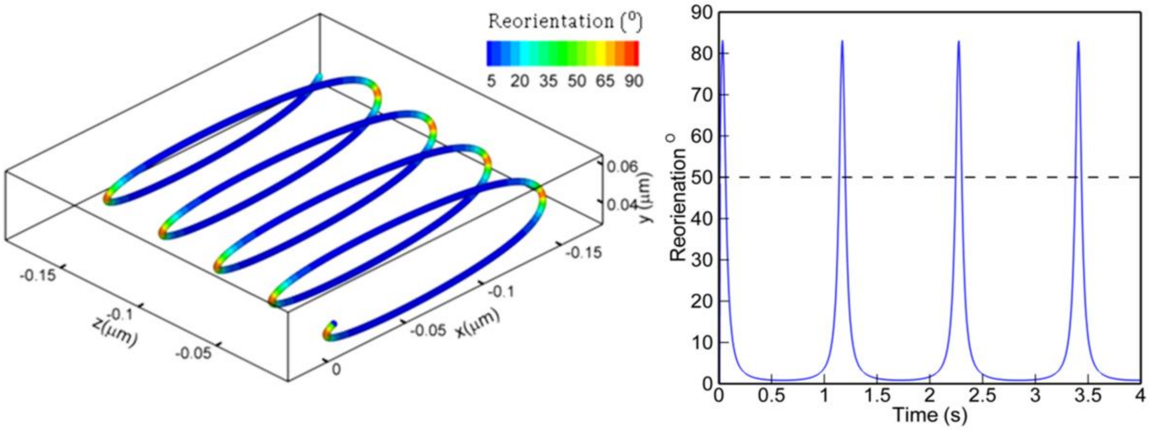

Fig. S7| (a) 3D Trajectory of a simulation of a smooth swimming *E. coli*. (Simulation conditions: cell swimming speed, 15 μm/s, cell aspect ratio, 10; and the shear, 30 s^{-1}. The initial conditions: $y = 5\mu m$, and $\phi(0) = \theta_z(0) = \frac{pi}{4}$). The trajectory is colorcoded by corresponding cell reorientations. (b) Cell reorientation over a time lapse of 1/15s. Dashed line: indicates the minimum reorientation angle that is considered as a tumble.

Fig. S7 shows the simulated trajectory and cell reorientation over 1/15 s. The simulation is conducted under the conditions (Table S1). The black dashed line in the figure indicates the minimum reorientation to be considered as a "pseudo" tumble (50°). The time lapse between two adjacent "pseudo" tumbles (time lapse of the re-orientation below 50°) is defined as mean run time and the inverse is considered as the characteristic tumble frequency, λ. When there is no "pseudo" tumbles detected, i.e. the cell reorientation over 1/15 s is below 50°, λ will be zero. For instance, the "pseudo" tumble frequency is measured as 0.962 s^{-1} in the example provided in Fig. S7. The example demonstrates anecdotally that when the flow shear is sufficiently large, the cell can indeed be reoriented owning to Jeffrey Orbit motion.

S2.3 Phase diagrams of "pseudo" tumbles

The results of simulation example show that Jeffrey Orbit motion of a prolong cell body (including flagella) at high flow shear can reorient itself with large angle to be considered as a "pseudo" tumble (Fig. S7). It has been shown by Jeffrey (1952) and many others that angular motion of the passive particle in a shear flow depends deterministically on flow shear, S, aspect ratio of particle, r_e, and initial cell orientation, $\vec{e}_b(0)$. In this section, we apply the developed simulations above

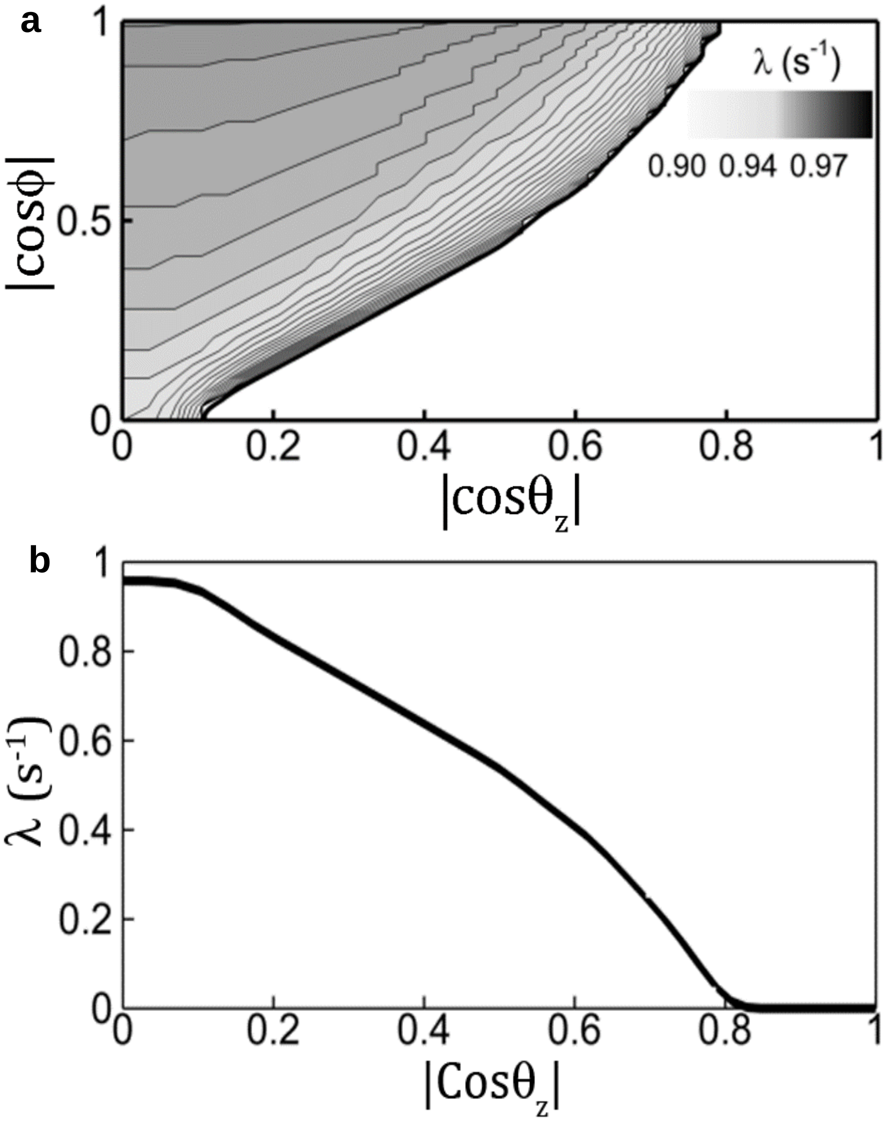

Fig. S8| (a) The phase distribution of the characteristic tumbling frequency, λ, with respect to initial c cell orientation, ϕ and θ_z; (b) Mean tumbling frequency, λ, averaged over those initial orientation vectores lying on a cone surface with θ_z as its major axis. The simulation is conducted at a constant flow shear of $S = 30\ s^{-1}$ and cell aspect ratio of 10.

(S.2.1) to explore the effects of S, r_e, and $\vec{e}_b$ on the characteristic tumbling frequency, λ, of a "pseudo" tumble.

Initial orientation on pseudo tumble: It has been shown that at a constant flow shear, S, the reorientation angle of *E. coli*, $\vec{e}_b(t)$, depends highly on the initial orientation of the cell, $\vec{e}_b(0)$, i.e. $\phi(0)$ and $\theta_z(0)$. The phase diagram of the characteristic tumbling frequency, $\lambda(\phi, \theta_z)$, is shown in Fig. S8a. The simulations are conducted using a constant flow shear, $S = 30\ s^{-1}$ and cell parameters listed in Table S1. The results shows that when the cell is oriented in the plane of flow shear (*x*-*y* plane, i.e. $\cos(\theta_z) = 0$ or $\theta_z = 90°$), λs reach its maximum values, whereas no pseudo-tumbles (e.g. $\lambda = 0$) are detected as the cell initially aligns in the normal direction to shear (e.g. $|\cos(\theta_z)| = 1$). Note that Fig. S8a contains a substantial area of $\lambda = 0$. The non-zero λ area reduces as the cell increasingly aligns in z direction. To examine the effect of angular orientation of θ_z on λ, we conditionally average λs over all ϕs with constant $|\cos\theta_z|$ (shown in Fig. S8b). The results reveals that the mean "pseudo" tumbling frequency, λ, reduces from its maximum when the cell aligns in the flow direction to zero when it aligns in the cross-flow direction. The cut-off angle yielding $\lambda = 0$ is $\theta_z = 36.8°$ (or $\theta_z = 143.2°$, $|cos\theta_z| = 0.8$). The results are expected

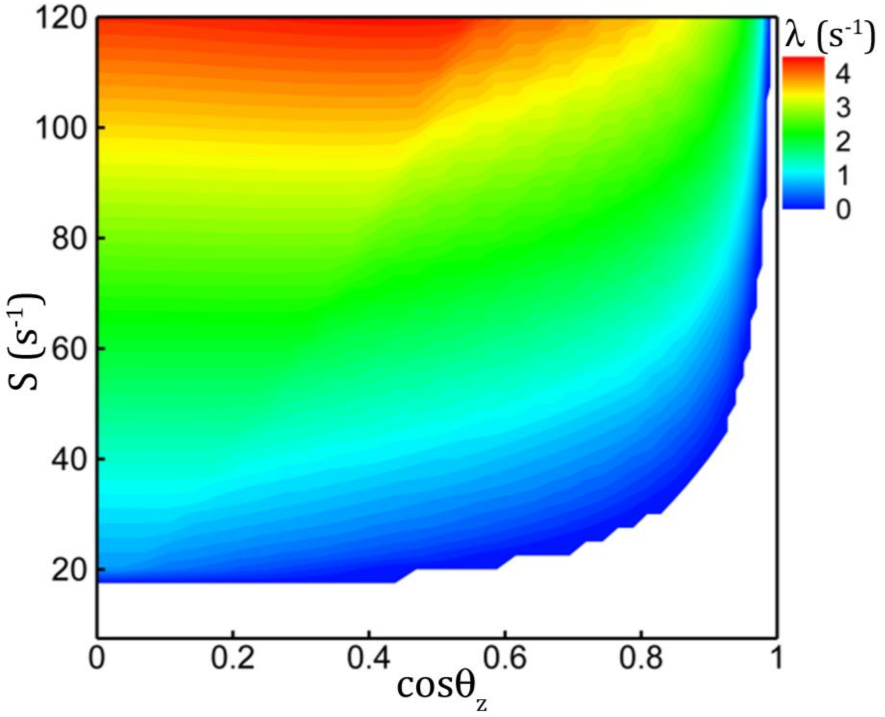

Fig. S9| The averaged pseudo-tumbling frequency vs the flow shear, S and the cosine of θ_z. The cell parameter used is listed in Table S1.

since the larger angle to the shear normal direction cause a faster reorientation of the cell by flow shear; whereas when the cell is aligned in the cross flow direction, the flow shear only rotates the cell around the fore-aft axis, which causes small change in the swimming direction. The results adequately explain the deviation of λ profile for the shear, $S = 30\ s^{-1}$ (Diamonds in Fig. 5b).

Critical flow shears causing "pseudo" tumbles: We perform the same analysis for the range of flow shear and compute the conditionally averaged frequency of "pseudo" tumbles at constant cell orientation to cross flow (z) direction, $\lambda(|cos\theta_z|)$. The simulation is conducted with cell parameters listed in Table S1. The results are presented in Fig. S10. As expected, the λs increase as the flow shear increases, since the viscous drag on the passive particle increases with flow shear and causes the period of the Jeffery Orbit shorter. Additionally, the critical shear-normal alignment angle, θ_z, reduces as the flow shear increases (boundary between non-zero and zero λ). Furthermore, Fig. S9 reveals that there is a critical flow shear below which no pseudo tumble may occur regardless of cell initial orientation. The critical flow shear is $S_{critical} = \sim 18\ s^{-1}$. This result corroborates well with the experimental observations (Fig. 5b) and our assertion that at low flow shear regime, Jeffery Orbit is not the mechanism to cause sudden change in bacterial swimming directions.

Critical flow shear vs particle parameter: To complete the analysis, we further compute the critical shear, $S_{critical}$ necessary to cause pseudo-tumble with respect to cell parameter, the effective cell aspect ratio (r_e). Fig. S10 shows how $S_{critical}$ changes with respect to r_e. The figure shows that critical shear, $S_{critical}$, decreases exponentially as r_e increases, i.e. the cell with larger r_e requires lesser flow shear, $S_{critical}$, to perform pseudo-tumbles. It can be observed that an asymptotic $S_{critical}$ exist, which suggests that pseudo-tumble can only occur above a minimum flow shear of 18 s^{-1}. This results further supports our assertion that conventional mechanism of cell re-orientation in a shear flow, namely Jeffery Orbit, can only be effective at high flow shear regime ($S > 18s^{-1}$); an alternative mechanism must be needed to explain the observation on the enhancement of tumbling motility (Figs. 2 & 3 in main text) at the lower flow shear regimes as well as a complimenting mechanism at higher flow shears.

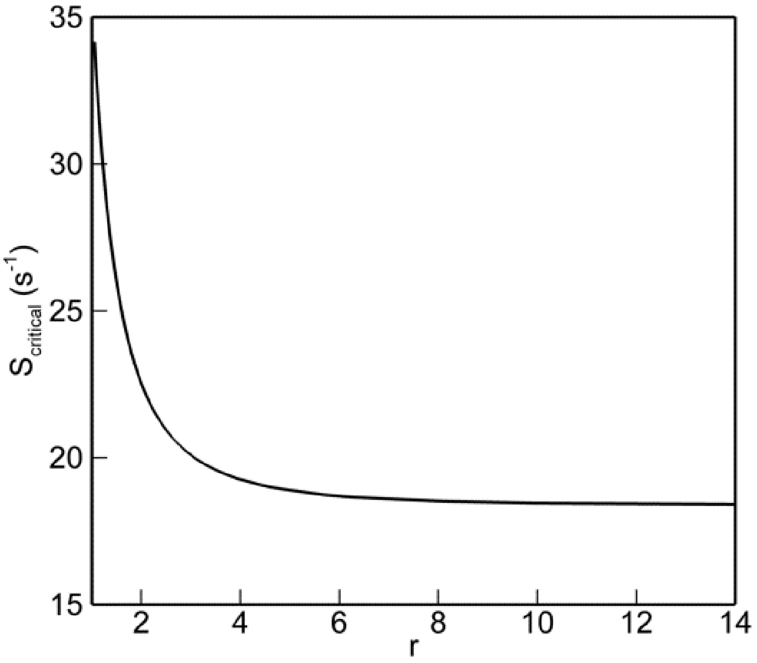

Fig. S10| The critical shear, $S_{critical}$, for pseudo-tumble vs. the effective cell aspect ratio,

S2.4. Further analysis to support shear induced unbundling mechanism

In this subsection, we provide further analysis to support our assertion that the flow shear acts in favor of bacterial flagellar unbundling and subsequently improve the tumbling motility near a solid surface, whereas it is otherwise suppressed in the quiescent condition[11]. Instead of performing full scale modeling, we will conduct analysis to answer the following two questions:

(i) What is the magnitude of the augmented unbundling stresses on flagella bundle during a shear accelerated unbundling event in comparison to those otherwise suppressed by hydrodynamic hindrance mechanism?

(ii) What is the critical shear rate at which the effect of shear accelerated unbundling and that of hydrodynamic hindrance mechanism balances each other?

Molaei *et al.* (2014) [11] have presented a conceptual model that a solid surface lengthens the timescale of flagella unbundling process, and subsequently causes an unsuccessful execution of a tumble, which effectively combines two consecutive runs into a long run. In that model, we use a drift velocity concept to model the process (S.3.2 in Ref [11]). In a nutshell, since the unbundling process can be estimated by the relative motion of flagella to its surrounding flow, namely drift

velocity (V). This developed framework (S.3.2 in Ref [11]) are applied here to answer the above questions:

Order of magnitude analysis: To address the question on how significant the shear induced unbundling stress in comparison to that of stress reduction due to hydrodynamic hindrance imposed by a solid surface, we compare the magnitudes of the drift velocity attributed directly to the flow shear and the surface suppression. Note that direct comparison of the drift velocity instead of stress is justified since the hydrodynamics is in Stokes flow regime.

Molaei et al. (2014) have related the runtime to the drift velocity of the flagella as

$$\frac{T_s^r}{T_b^r} \approx 1 - \frac{V_s^d - V_b^d}{V_b^d}, \quad \text{(S12)}$$

where T is the mean runtime and V^d is the drift velocity. Hereinafter, subscript b and s denote bulk and surface respectively. The reduction of drift velocity near a surface in a quiescent condition in comparison to that in bulk ($\Delta V_{hind} = V_s^d - V_b^d$) is estimated then compare it directly with the increase of drift velocity in the presence of flow shear, ΔV_{shear}, in comparison to that in the uniform advection flow condition. Molaei *et al.* (2014) have shown that the drift velocity between two flagella can be further related to the external cross flow, u_f, (S.3.2 in Ref [11]) as

$$\frac{V_s^d - V_b^d}{V_b^d} \approx \frac{u_{f,s} - u_{f,b}}{u_{f,b}}, \quad \text{(S13)}$$

and can be modeled and linearized to $C_1 \left(\frac{y}{L_c}\right)^{-1}$, which is the first term of Eqn. S19 in SI of Ref [11], where y is the distance to the surface and L_c the total length of the cell including the flagella. For the given physiological parameters of an AW405 bacterium, the constant is estimated as $C_1 \approx 0.4$ (following the same procedure described in S.3.4 in SI of Ref [11]). The reduction of the drift velocity can then be found in the simple form as

$$\Delta V_{hind} \approx C \left(\frac{y}{L_c}\right)^{-1} u_{f,b} \quad \text{(S14)}$$

In a quiescent condition, the relative flow around a rotating flagellum, $u_{f,b}$, is simply the rotational speed of the filament, i.e. $u_{f,b} = \omega_f r_f$, where ω_f is the angular rotation of the filament and r_f is the effective radius of the filament. Note that r_f is the radius of a helical filament (e.g. 0.2 μm), not the radius of a flagellum (e.g. 10nm). For an *E. coli* cell, the physiological parameters are: $L_c \approx 10\ \mu m$, $\omega_f \approx 100\ Hz$, and $r_f \approx 0.2\ \mu m$. When the cell swims in a near-surface region of $y = 5 - 20\ \mu m$, one can obtain $\Delta V_{hind} \approx 4 - 16\ \mu m/s$.

In a shear flow, the augmented drift velocity between an unbundling flagellum and the flagella bundle can be described as

$$\Delta V_{shear} \approx \gamma r_b cos\theta_z, \quad \text{(S15)}$$

where γ is the local flow shear; r_b is the radius of the cell body; and θ_z is the angle between the swimming and the cross-flow direction. For a flow in a micro-channel, the near surface flow shear is determined as $\gamma = \left(1 - \frac{2y}{H}\right) S$, where S is shear at surface and H the height of the micro-channel (e.g. $H = 200\mu m$). For an *E. coli* of $r_b = 0.5\ \mu m$ swimming in the near surface region with a surface flow shear of $S = 30\ s^{-1}$, the augmented drift velocity is $\Delta V_{shear} \approx 12 - 14\ \mu m/s$. This analysis demonstrates that the effect of flow shear is significant enough to mitigate the effect by surface suppression on unbundling process. Note that the magnitude of ΔV_{shear} and ΔV_{hind} are closely comparable in magnitudes and ranges.

Critical Shear: By equating Eqn. S14 and S15, one can obtain a critical shear rate, $S_{critical}$, at which the effect of flow shear on unbundling balances that of surface hydrodynamic hindrance:

$$S_{critical} \approx C_1 L_c \omega_f r_f H/[y(H - 2y)r_b] \quad \text{(S16)}$$

It shows that $S_{critical}$ depends on the distance to the surface. One can further integrate Eqn. S16 along the distance to the wall (y) over the near surface region ($0.5 \leq\ y \leq 20\mu m$) excluding one radius of cell body ($r_b = 0.5\mu m$) to obtain a mean critical shear, $\bar{S}_{critical} = \int_{0.5\mu m}^{20\mu m} C_1 L_c \omega_f r_f H/[y(H - 2y)r_b]dy$. The approximated $\bar{S}_{critical}$ for an AW405 bacterium is $31.8\ s^{-1}$ that agrees well with the experimental observation of $30\ s^{-1}$(Fig. 5b).

References

1 Berke, A. P., Turner, L., Berg, H. C. & Lauga, E. Hydrodynamic attraction of swimming microorganisms by surfaces. 101 (2008).

2 Duffy, D. C., McDonald, J. C., Schueller, O. J. A. & Whitesides, G. M. Rapid prototyping of microfluidic systems in poly(dimethylsiloxane). *Anal. Chem.* 70, 4974-4984 (1998).

3 Chengala, A., Hondzo, M. & Sheng, J. Microalga propels along vorticity direction in a shear flow. *Phys. Rev. E* 87 (2013).

4 Molaei, M. & Sheng, J. Imaging bacterial 3D motion using digital in-line holographic microscopy and correlation-based de-noising algorithm. *Opt. Express* 22, 32119-32137 (2014).

5 Sheng, J., Malkiel, E. & Katz, J. Digital holographic microscope for measuring three-dimensional particle distributions and motions. *Appl. Optics* 45, 3893-3901 (2006).

6 Sheng, J., Malkiel, E. & Katz, J. Using digital holographic microscopy for simultaneous measurements of 3D near wall velocity and wall shear stress in a turbulent boundary layer. 45, 1023-1035 (2008).

7 Sheng, J., Malkiel, E. & Katz, J. Buffer layer structures associated with extreme wall stress events in a smooth wall turbulent boundary layer. 633, 17-60 (2009).

8 Sheng, J. *et al.* Digital holographic microscopy reveals prey-induced changes in swimming behavior of predatory dinoflagellates. 104, 17512-17517 (2007).

9 Sheng, J. *et al.* Prey-induced changes in swimming behavior of predatory dinoflagellates. 43, 25-25 (2007).

10 Sheng, J., Malkiel, E., Katz, J., Adolf, J. E. & Place, A. R. A dinoflagellate exploits toxins to immobilize prey prior to ingestion. 107, 2082-2087 (2010).

11 Molaei, M., Barry, M., Stocker, R. & Sheng, J. Failed Escape: Solid Surfaces Prevent Tumbling of Escherichia coli. *Phys. Rev. Lett.* 113 (2014).

12 Marcos, Fu, H. C., Powers, T. R. & Stocker, R. Bacterial rheotaxis. 109, 4780-4785 (2012).

13 Berg, H. C. & Brown, D. A. Chemotaxis in Escherichia coli Analysed by Three-dimensional Tracking. 239, 500-504 (1972).

14 Zottl, A. & Stark, H. Nonlinear Dynamics of a Microswimmer in Poiseuille Flow. *Phys. Rev. Lett.* 108 (2012).

15 Zottl, A. & Stark, H. Periodic and quasiperiodic motion of an elongated microswimmer in Poiseuille flow. *Eur. Phys. J. E* 36 (2013).

16 Jeffery, G. B. The Motion of Ellipsoidal Particles Immeresed in a Viscous Fluid. 102, 161-179 (1922).
